# Mechanistic Modeling of *Aedes aegypti* Mosquito Habitats for Climate‐Informed Dengue Forecasting

**DOI:** 10.1029/2025GH001376

**Published:** 2025-09-16

**Authors:** C. N. Yasanayake, B. F. Zaitchik, A. Gnanadesikan, L. M. Gardner, A. Shet

**Affiliations:** ^1^ Department of Earth & Planetary Sciences Johns Hopkins University Baltimore MD USA; ^2^ Department of Civil & Systems Engineering Johns Hopkins University Baltimore MD USA; ^3^ Department of Epidemiology Johns Hopkins Bloomberg School of Public Health Baltimore MD USA; ^4^ Department of International Health Johns Hopkins Bloomberg School of Public Health Baltimore MD USA

**Keywords:** dengue, climate variability, mechanistic modeling, Aedes aegypti, vector‐borne disease, vector habitats

## Abstract

The mosquito‐borne disease dengue is sensitive to climate, in part because of the influence climate has on breeding habitats of dengue's *Aedes* mosquito vectors. Dengue risk assessment models currently leverage climate‐dengue *statistical* associations, yet what remain understudied are the *mechanistic* pathways that yield different statistical relationships in different locations. We hypothesize that elucidating the mechanisms by which spatiotemporal variability in climate influences dengue incidence will improve dengue dynamics predictions across climatically distinct locations and beyond dengue's well‐known seasonal cycles. We test this hypothesis by investigating a key pathway in the climate‐dengue process chain: climate impacts on *Aedes* breeding habitats. We have implemented a mechanistic modeling pipeline that simulates climatic influence on habitat water dynamics and thereby on relative population size of the vector. We use this modeling pipeline, driven by meteorological data, to simulate monthly *Aedes* populations for three climatically distinct cities in Sri Lanka. We find that simulated vector abundance is plausibly associated with climate conditions and that climate drivers of vector abundance vary among locations. Moreover, tercile‐tercile comparisons of dengue incidence against model variables indicate that risk assessments based on predicted vector abundance perform similarly to those based on meteorology alone—the signal of weather variability and its relationship to dengue propagates through the modeling pipeline. These results justify future testing of this modeling pipeline within a dengue risk assessment framework, where its process‐based structure may be leveraged to guide proactive dengue control efforts in high‐risk years and to simulate impacts of future climate conditions on dengue dynamics.

## Plain Language Summary

While it is well known that climate influences incidence of the mosquito‐borne disease dengue, our ability to use climate data (e.g., air temperature and rainfall) to predict dengue risk and inform public health responses is hampered by limited quantitative understanding of the causal pathways by which climate impacts dengue. In this work we improve our understanding of one such pathway: how much of the climatic impact on dengue is explained by climate impacts on the mosquitoes' aquatic breeding habitats? These habitats—water‐filled containers such as water tanks and flowerpots—host the mosquito eggs, larvae, and pupae, which require an aquatic environment at a suitable temperature. Using a specialized mathematical model, we simulated how climate conditions (primarily air temperature and rainfall) influence water in mosquito breeding habitats and how this affects mosquito abundance. We tested the model in three Sri Lankan cities with different climates and found that predictions of dengue risk based on mosquito abundance were similar in effectiveness to predictions based directly on weather data. These results suggest that our model's capacity to refine dengue risk predictions using climate data warrants further testing, with potential to inform early warning systems and guide timely and targeted disease prevention efforts.

## Key Points

- Dengue risk is influenced by climate—which impacts *Aedes aegypti* mosquito breeding sites—but its mechanistic pathways remain understudied
- We used a mechanistic modeling pipeline to simulate climate impacts on mosquito abundance in three climatically diverse Sri Lankan cities
- The model aligns with weather‐based dengue predictions, showing potential to refine forecasts and guide interventions as climate changes

## 1 Introduction

The mosquito‐borne disease dengue is one of the most rapidly growing infectious diseases in the world. Once known as breakbone fever due to the extreme pain it can cause (Guzman et al., 2016; Wong et al., 2014), dengue has become a growing public health concern in recent decades given an alarming increase in not only the incidence of new cases, but also in the severity of the disease and its geographical extent (Brady et al., 2012; Gubler, 2004; Tapia‐Conyer et al., 2009; Van Kleef et al., 2011). It is estimated that about half the global population is at risk due to living in the tropical and subtropical regions that are climatically favorable for the mosquito vectors that transmit the dengue virus (Bhatt et al., 2013; Brady et al., 2012; Messina et al., 2019). Dengue's dramatic rise has myriad reasons, but is attributed in part to rapid urbanization, human population growth, and increased global connectivity, all of which foster greater contact—and therefore higher dengue virus transmission—among human and mosquito populations across the world (Glaesser et al., 2017; Guzman & Harris, 2015; Huang et al., 2012; Lopez et al., 2016; Quam et al., 2015; Sessions et al., 2013; Struchiner et al., 2015; Wilder‐Smith et al., 2010, 2019). Moreover, global climate change is expected to further exacerbate the burden of dengue in years to come, as increasing temperatures will expand the geographical range of the mosquito vectors and put new human populations at risk (Chang et al., 2014; Messina et al., 2019; Ryan et al., 2019; Shepard et al., 2011; Wilder‐Smith et al., 2019).

The growing threat of dengue has motivated the development of climate‐informed early warning systems. Such systems have shown some promise in detecting emerging outbreaks (e.g., Hussain‐Alkhateeb et al., 2018), yet dengue prediction models still have ample room for further development and improvement of their forecasting skill (Leung et al., 2023). In particular: can a more nuanced integration of climate information into such a system improve prediction of dengue and inform targeted interventions? At present, most dengue prediction models incorporate climate data in the form of statistical associations (e.g., correlations between amount of rainfall and dengue incidence) (Leung et al., 2023). However, this approach neglects the complexities of the numerous underlying process chains via which climate impacts dengue. For example, larva and pupae of *Aedes aegypti*, the primary vector for dengue virus in most of the tropics, optimally develop and survive within a temperature range of approximately 24–34°C (Bar‐Zeev, 1958; Eisen et al., 2014; Farjana et al., 2012; Kamimura et al., 2002; Mohammed & Chadee, 2011; Padmanabha et al., 2011; Richardson et al., 2011; Rueda et al., 1990; Tun‐Lin et al., 2000) and adult females take more frequent blood meals at higher temperatures (Scott et al., 2000). Rainfall, when in moderate amounts, can create pools of still water that can serve as breeding sites, encouraging greater vector abundance (Kakarla et al., 2019; Morin et al., 2013). However high intensity rainfall can suppress vector abundance by washing out breeding sites and the eggs, larvae, and pupae that live in them (Morin et al., 2013; Xiang et al., 2017; Watson et al., 2007). High relative humidity (∼80%) is conducive to greater oviposition rates (Canyon et al., 1999; Costa et al., 2010) and longer lifespans (Costa et al., 2010; Ramachandran et al., 2016; Reiskind & Lounibos, 2009), presumably due to lower environmental stress from dehydration (Costa et al., 2010). Beyond its influence on vectors, climate may also impact dengue infection rates by altering human mobility patterns that in turn influence the frequency of interactions between humans and the mosquito vectors.

In this work we explore the value of mechanistic modeling for dengue forecast applications, assembling a modeling pipeline that simulates one critical set of processes connecting climate and dengue: the impact of climate on mosquito breeding habitat suitability and thereby on mosquito population size. The climatic sensitivity of breeding habitats is important because the microclimate experienced by mosquito eggs, larvae, and pupae in the breeding habitats (typically water‐filled artificial containers) can be quite different from the climate conditions captured by conventional meteorological data (e.g., 2‐m air temperature). We simulate these breeding habitat microclimates based on different years of meteorological data, allowing us to analyze how interannual variability in climate propagates through the modeling pipeline. If interannual variability propagates systematically through the modeling pipeline, then the modeling outputs may aid dengue predictions beyond the known seasonal patterns of disease incidence and the process‐based modeling pipeline might therefore be used to inform intervention strategies in high‐risk years.

**Figure 1:**
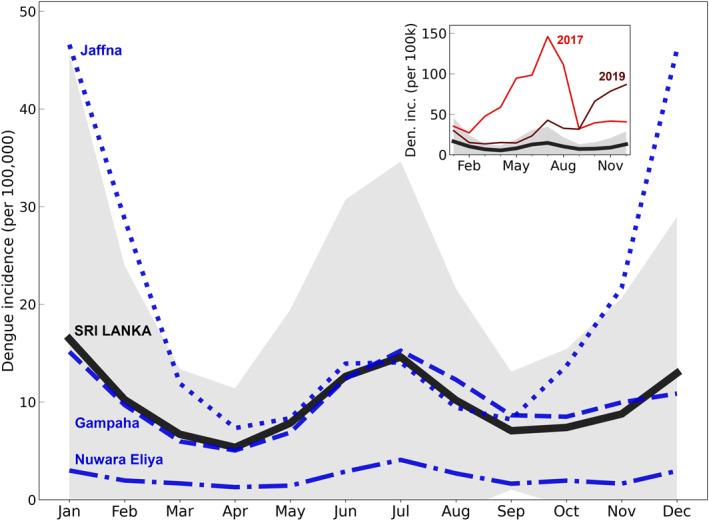
Average monthly reported dengue incidence in non‐outbreak years for all of Sri Lanka (solid black line), Jaffna District (blue dotted line), Gampaha District (in which the city of Negombo is located; blue dashed line), and Nuwara Eliya District (blue dash‐dotted line) (Sri Lanka Ministry of Health—Epidemiology Unit, 2025; Central Bank of Sri Lanka, 2023; Sri Lanka Department of Census and Statistics, 2025). This data is averaged over the years 2007–2020, excluding the outbreak years 2017 and 2019. The gray shading shows the one standard deviation interval for the all‐Sri Lanka data. **[inset]** The same averaged all‐Sri Lanka dengue incidence for non‐outbreak years as shown in the main plot (solid black line and gray shading) alongside the all‐Sri Lanka dengue incidence in the outbreak years 2017 and 2019 (solid red lines).

We focus this work on the tropical country of Sri Lanka, which is heavily dengue‐impacted and has shown clear associations between climate and dengue. Dengue has been endemic in Sri Lanka since the 1960s, but in recent decades the epidemiological landscape has shifted: there has been a 20‐fold increase in dengue incidence from the year 2000–2012, a greater share of infections and deaths now occur in older adults, and recent years have seen large island‐wide outbreaks, namely in 2017 and 2019 (Malavige et al., 2021; Tissera et al., 2020). While these outbreaks are attributed primarily to non‐climate factors (i.e., circulation of a dengue serotype to which the population has low levels of immunity) (Tissera et al., 2020), there is notable interannual variability in dengue incidence even among non‐outbreak years that may be attributable to climatic variability (Figure 1). There are also well‐known seasonal cycles to dengue incidence in Sri Lanka that suggest strong, region‐specific climate sensitivities, particularly to rainfall (Hooshyar et al., 2020; Prabodanie et al., 2020). To capture these regional differences our work focuses on three Sri Lankan cities in distinct climate zones: Negombo, a coastal southwestern city with high temperature, high rainfall, and high dengue incidence; Jaffna, a coastal northern city with high temperature, low rainfall, and high dengue incidence; and Nuwara Eliya, a central highlands city with low temperature, high rainfall, and low dengue incidence.

The remainder of this paper describes our simulations of climate impacts on dengue vector breeding habitats for each of the cities Negombo, Jaffna, and Nuwara Eliya, investigating how interannual variability in climate is transformed into variability in habitat suitability and then into variability in vector population size. Section 2 describes the meteorological and dengue case data sets used in this work and outlines the structure, specifications, and inherent assumptions of the modeling pipeline. Section 3 analyzes the propagation of climate signals through the modeling pipeline and considers associations between observed dengue incidence and the variables produced by the model (e.g., mosquito adult population size). We conclude in Section 4 with key takeaways from this work.

## 2 Data and Methods

### 2.1 Data

#### 2.1.1 Meteorological Data

We simulate container water dynamics using the model WHATCH’EM, which requires inputs of precipitation, air temperature, and relative humidity and has optional inputs of cloud cover and soil temperature. In addition, vapor pressure deficit was used as an input for calculating vector survival (mortality due to desiccation). These data were obtained from gridded global products, from which we chose the data grid cells nearest to our three locations of interest in Sri Lanka: the cities Negombo, Nuwara Eliya, and Jaffna. All data are hourly and span the years 2001–2020. However we only use a subset of this data in Section 3.2, where we compare model results against dengue case data for non‐outbreak years (2007–2020, excluding outbreak years 2017 and 2019). These data are detailed in Table 1.

**Table 1:** Meteorological Data Used in This Study

Meteorological field	Data source	Variable name
Precipitation*	IMERG	precipitationCal
Air temperature (2‐m)**	MERRA‐2	T2M
Relative humidity (2‐m)	MERRA‐2 (derived)	Derived from… 2‐m air temperature (T2M), 2‐m specific humidity (QV2M), and surface pressure (PS)
Cloud area fraction (low clouds)	MERRA‐2	CLDLOW
Cloud area fraction (middle clouds)	MERRA‐2	CLDMID
Cloud area fraction (high clouds)	MERRA‐2	CLDHGH
Soil temperature (0–0.0988 m)**	MERRA‐2	TSOIL1
Vapor pressure deficit	MERRA‐2 (derived)	Derived from… 2‐m air temperature (T2M)**, 2‐m relative humidity (derived; see its entry in this table)

*Half‐hourly data resampled to hourly. **For Nuwara Eliya only, data was bias‐corrected based on a linear regression of T2M daily averages against Global Surface Summary of the Day observations of daily average air temperatures.

All variables except for precipitation (i.e., air temperature, relative humidity, cloud cover, soil temperature, vapor pressure deficit) were obtained from the Modern‐Era Retrospective analysis for Research and Applications 2 (MERRA‐2) (Global Modeling and Assimilation Office, 2015a, 2015b, 2015c). MERRA‐2 is produced by the NASA Global Modeling and Assimilation Office using the Goddard Earth Observing System Model version 5.12.4. It is a global data product with a spatial resolution of 0.5° × 0.625° and an hourly temporal resolution.

Precipitation data were obtained from the Integrated Multi‐Satellite Retrievals for GPM (IMERG) Final Run (Huffman et al., 2023), which merges microwave and infrared satellite measurements with gauge observations to produce a global data product with a spatial resolution of 0.1° × 0.1° and a half‐hourly temporal resolution. For this work we resampled the half‐hourly data to hourly resolution.

These two data products, MERRA‐2 and IMERG, were chosen primarily because of their broad spatial and temporal coverage and because of the need for hourly temporal resolution (to match the hourly cadence of the WHATCH’EM model's container dynamics simulations). Using ground‐based station data instead would be ideal for local accuracy—to avoid the uncertainty and error that satellite‐based and reanalysis products have relative to observations (e.g., Bosilovich et al., 2008; Gehne et al., 2016) —but station data in Sri Lanka is inconsistent in spatial and temporal coverage due to the sparseness of stations in the northern and eastern regions of the country and due to gaps in station data records during periods of conflict (Jayawardena et al., 2020; Wijemannage et al., 2016). We considered other satellite‐derived precipitation estimates such as the Climate Hazards Group Infrared Precipitation with Station (CHIRPS v2.0) product (Funk et al., 2015), which outperforms IMERG Early Run for Sri Lanka (Bandara et al., 2022), but we ultimately selected IMERG because of our need for hourly precipitation estimates.

While we use MERRA‐2 and IMERG because they have these advantages relative to ground‐based station data and other satellite‐based and reanalysis data sets, it would be prudent to assess and mitigate their biases as much as we can. We therefore compared the MERRA‐2 and IMERG data to available Global Surface Summary of the Day (GSOD) weather stations (NOAA National Centers of Environmental Information, 1999) for each of the three locations (Negombo, Nuwara Eliya, Jaffna) and for the variables that had corresponding station data: precipitation, air temperature, and relative humidity. We found that the reanalysis and observation data sufficiently agreed for Negombo and Jaffna. However for Nuwara Eliya, our most mountainous site, the MERRA‐2 air temperature was notably higher than station data, so we bias‐corrected the MERRA‐2 air temperatures based on a linear regression of MERRA‐2 versus GSOD station daily average air temperatures (for more details, see Text S1 in Supporting Information S1). The Nuwara Eliya MERRA‐2 soil temperature values were also high enough to suggest a positive temperature bias. Since we did not have ground‐based soil temperature to perform an analogous bias correction, we bias‐corrected the MERRA‐2 soil temperatures based on the same linear regression of MERRA‐2 versus weather station daily average air temperatures (i.e., we assumed that the mathematical relationship between MERRA‐2 air temperature and GSOD air temperature was the same as the relationship between MERRA‐2 soil temperature and the actual soil temperature).

It should be acknowledged that the MERRA‐2 and IMERG data sets cannot perfectly describe the meteorological conditions of Negombo, Nuwara Eliya, and Jaffna. We represent each city's meteorology using the nearest MERRA‐2 and IMERG grid cell, and these grid cells are quite large (MERRA‐2: 0.5° × 0.625°; IMERG: 0.1° × 0.1°). Each cell may therefore encompass an area that is larger than, or spatially offset from, the cities themselves. Moreover, cities often have significant variations in climate over small spatial scales, otherwise known as microclimates (e.g., cooler and more humid conditions under tree canopy), which are simply not captured in the large‐scale view provided by MERRA‐2 and IMERG. These small‐scale nuances of urban climate can be quite relevant for mosquito habitability and mosquito‐borne disease incidence (e.g., Romeo‐Aznar et al., 2024; Wimberly et al., 2020). In this work we must leave these small‐scale complexities unexplored, and we instead focus on how the distinct, large‐scale meteorological patterns at these locations translate to characteristic differences in container habitat suitability. We encourage future work to expand on these results by taking into consideration the role of urban microclimates.

#### 2.1.2 Dengue Incidence Data

The work presented here focuses on the sensitivity of *Ae. aegypti* breeding site and survival models to climate variability—we do not attempt dengue forecasts. Nevertheless, we do use dengue incidence data to explore associations between our simulation outputs and reported dengue incidence as an indicator of the potential utility of the system for future dengue prediction modeling.

We computed dengue incidence based on dengue case data obtained from the weekly epidemiological reports published by the Sri Lanka Ministry of Health (Sri Lanka Ministry of Health – Epidemiology Unit, 2025). These data are published for each of Sri Lanka's 26 Regional Directorates of Health Services (RDHS). For this study we used data for 2007–2020 from the RDHSs at our three locations of interest (Negombo—RDHS Gampaha, Nuwara Eliya—RDHS Nuwara Eliya, Jaffna—RDHS Jaffna). We summed the weekly data to a monthly cadence to match our month‐long model runs. We also converted dengue case counts to dengue incidence (cases per 100,000 individuals) by using annual district‐level population data (Central Bank of Sri Lanka, 2023; Sri Lanka Department of Census and Statistics, 2025), allowing clearer comparisons across these three locations of interest despite their different population sizes.

There are a few caveats to the use of this dengue case data that deserve mention. For one, we have said that our three locations of interest are the cities of Negombo, Nuwara Eliya, and Jaffna, but this dengue data is at the larger‐scale district level. So we are implicitly assuming that dengue incidence in each city tracks with dengue incidence in the district as a whole, which we consider reasonable since these three cities are major urban centers in their respective districts. We also expect this discrepancy to minimally impact our work because we climatically represent these cities using meteorological data for areas larger than the cities themselves (see discussion of MERRA‐2/IMERG grid cell sizes in Section 2.1.1).

A key limitation of this data set (and any disease case data set, for that matter) is that it may not capture all dengue incidence. Some people who are infected may never seek healthcare and may never have their case recorded. This is particularly likely for a disease like dengue where milder cases do not require hospitalization and have nonspecific symptoms (e.g., fever and headache). Moreover, the fraction of cases that are recorded may change from location to location and over time (e.g., due to differing access to medical care or differing diagnostic methods). Unfortunately, we do not know what fraction of all dengue cases are captured in this data and—more importantly for this work—whether this fraction is the same across our three locations of interest and over our timespan of interest. We make an assumption that this fraction is constant (i.e., that we can compare dengue incidence values across locations and over time), but this may not be true and should be considered in interpreting our results.

Another limitation of the dengue data is the potential for discrepancy between where a given person is infected with dengue and where they seek care. In other words, cases recorded in a given district do not necessarily represent infections that occurred in that district if people are seeking healthcare across district lines. The same is true for *when* dengue cases occur versus when they're recorded, as it may take several days after infection for symptoms to develop and for a person to seek medical attention (note that dengue virus incubation time is approximately 3–10 days (Chan & Johansson, 2012)). For this work we are assuming that the dengue case data reasonably reflects cases within the district boundaries, and in interpreting our results we keep in mind that there is a lag between when a dengue infection occurs and when it is recorded.

An additional nuance is that the dengue data is gathered at the district level, but the modeling we do in this work is based on meteorology from gridded data products whose grids do not align with district boundaries (Table 1). Therefore, when we compare our simulation outputs to reported dengue incidence we are not necessarily considering dengue infections that occurred under the same meteorological conditions used to run the model. However we expect this to be a minor source of error, particularly given that the grid cells of the meteorological data are comparable in size to the districts we consider.

### 2.2 Methods

#### 2.2.1 Modeling Pipeline Overview

**Figure 2:**
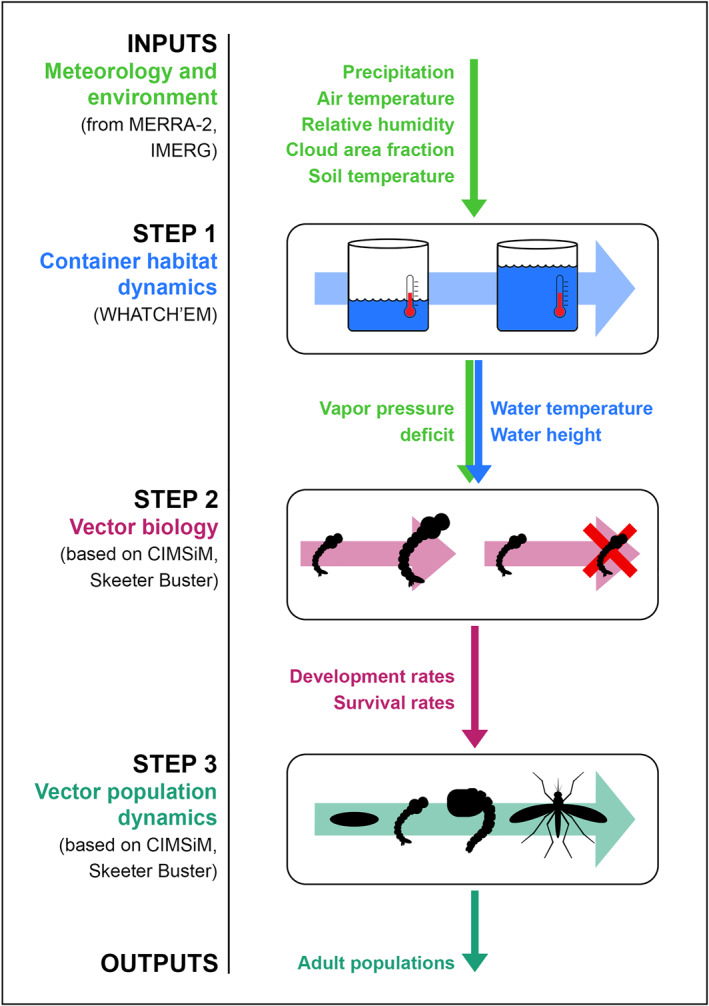
The modeling pipeline assembled for this work, which simulates climatic impacts on *Aedes aegypti* populations via their container breeding habitats. The pipeline has three modeling steps: (1) container habitat dynamics modeled using WHATCH’EM (Steinhoff et al., 2016), (2) vector biology modeled based on CIMSiM (Focks et al., 1993) and Skeeter Buster (Magori et al., 2009), and (3) vector population dynamics modeled based on CIMSiM and Skeeter Buster.

Our modeling pipeline consists of three steps, as depicted in Figure 2.

The first step is the WHATCH’EM model (Steinhoff et al., 2016), an energy balance model that simulates climatic impacts on the container habitats of *Ae. aegypti*. This modeling step translates meteorological variable inputs (primarily precipitation, air temperature, and relative humidity) into container water dynamics outputs (water height and water temperature).

The second step of the modeling pipeline is a set of vector biology equations describing development and survival of *Ae. aegypti* for each of its immature stages: egg, larva, and pupa. These vector biology equations have been used previously in mosquito population dynamics models CIMSiM (Focks et al., 1993) and Skeeter Buster (Magori et al., 2009). This modeling step translates container habitat conditions (water temperature, whether the container is dry, and vapor pressure deficit) into hourly development rate and daily survival rate for each immature life stage of *Ae. aegypti*.

The third step of the modeling pipeline is a population dynamics model based on CIMSiM and Skeeter Buster that simulates the growth of an initial population of *Ae. aegypti* immatures over the course of a month in a model container habitat. This modeling step translates *Ae. aegypti* development and survival rates into a population size of mosquitoes at each life stage (eggs, larvae, pupae, adults). This population size is not intended as an estimate of actual mosquito population size in each of these cities. Instead, it is best interpreted as a *relative* measure of environmental suitability for mosquito population growth.

We describe these three steps of the model pipeline in further detail in the following sections.

#### 2.2.2 Step 1—Container Habitat Water Dynamics

We use the WHATCH’EM energy balance container model (Steinhoff et al., 2016) to simulate climatic impacts on *Ae. aegypti*'s aquatic breeding habitats: the water‐filled artificial containers that host the eggs, larvae, and pupae. The inputs to WHATCH’EM are timeseries of meteorological data, which the model uses alongside user‐specified container parameters (Table S1 in Supporting Information S1) and location parameters (Table S2 in Supporting Information S1) to simulate the hourly temporal evolution of water height and water temperature within the container. The two model outputs, water height and water temperature, are critical for determining the container habitat's suitability for *Ae. aegypti* eggs, larvae, and pupae, as explored in detail in the next step of the modeling pipeline (see Section 2.2.3).

We have chosen to use WHATCH’EM for modeling container water properties because of its physics‐based approach, which contrasts with the empirical approaches employed by other *Ae. aegypti* habitat modeling work (e.g., Focks et al., 1993; Magori et al., 2009). WHATCH’EM's approach generally better estimates water height and water temperature as it better captures the nonlinear interactions between meteorological variability and container water dynamics. This has been demonstrated by validation experiments of container water dynamics in climatically distinct regions of Mexico (Steinhoff et al., 2016).

Container breeding sites are subject to human management—activities that result in containers being moved, emptied, filled, or otherwise changed from the unmanaged state can all have a dominant impact on the evolution of breeding microclimates. Indeed, an advantage of mechanistic models like WHATCH’EM is that they can be used to simulate the sensitivity of breeding sites to management. However, here we focus on the potential influence of climate on unmanaged containers. To do this, we run the model 1 month at a time, initiating water height at 10% of the container height (36.8 mm of water in a 368 mm tall container) and running the simulation for a full month. We do this for every month from 2001 to 2020. A month‐long run is long enough to capture the vector's aquatic lifecycle stages from oviposition to emergence as an adult (timing varies, but typically at least ∼10 days (Christophers, 1960)).

By making these modeling choices (month‐long runs, 10% initial water height, no manual filling) we simulate the time evolution of a container that begins as a viable habitat and has the potential to remain a viable habitat for the full duration of the model run.

We have also chosen to simulate one container type (i.e., a single set of container parameters, as described in Table S1 in Supporting Information S1). Rather than fully employing WHATCH’EM's capacity for container customization, we simulate a single “intermediate” container (e.g., the container color is gray rather than white or black; the container is in half shade rather than full shade or full sun). By choosing these intermediate container parameters we aim to minimize the discrepancy between our modeled container water dynamics and the dynamics in the actual container types that serve as vector habitats. We use this simple scheme because there is limited data on actual container types and we cannot accurately assess the real‐world container landscape in terms of physical properties of the containers, their quantity, their spatial distribution, and how these factors vary over time. This is a notable limitation that must be kept in mind when interpreting the results of this work. A container's physical properties will mediate temperature (e.g., a black container will attain higher temperatures than a white container) and temperature variability (e.g., a smaller container will experience greater temperature fluctuations than a larger container filled with the same fraction of water). Moreover, a container's physical location will determine the microclimate it experiences and its proximity to humans (both of which are relevant for gauging associated dengue risk).

#### 2.2.3 Step 2—Vector Biology

**Figure 3:**
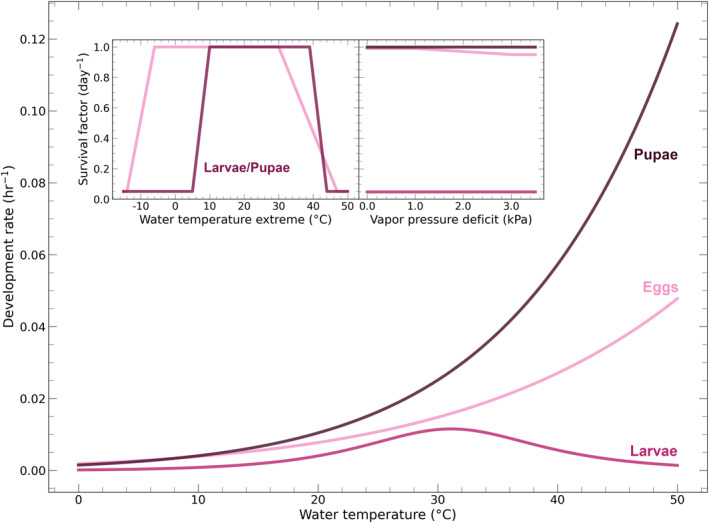
Hourly development rates and daily (temperature‐ and desiccation‐based) survival factors for eggs, larvae, and pupae, as used in Focks et al. (1993). Note that the desiccation‐based survival factors are only applicable for dry containers. Also, the desiccation‐based egg survival factor shown here is specifically for containers that are at least 15% shaded (as in this work, where shading is 50%).

We simulate the impact of container habitat conditions on the aquatic, immature stages of *Ae. aegypti* (eggs, larvae, pupae) using equations that describe *Ae. aegypti* development rates and survival factors. The development rates—dependent on water temperature—determine how quickly the eggs, larvae, and pupae progress from one stage to the next: eggs typically mature in 2–3 days, larvae in 4–5 days, and pupae in about 2 days (Carvalho & Moreira, 2017). The survival factors—dependent on water temperature extremes and, in dry containers where the immature stages are susceptible to desiccation, vapor pressure deficit—determine whether the eggs, larvae, and pupae survive each day of the simulation. These equations are shown graphically here (Figure 3) and in analytic form in (Text S3 in Supporting Information S1). We use these equations because they have been used previously in mosquito population dynamics models CIMSiM (Focks et al., 1993) and Skeeter Buster (Magori et al., 2009). Note that, unlike these other models, we do not model development and survival for adults and only focus on the immature stages: eggs, larvae, and pupae. This is because our modeling pipeline is particularly focused on how climatic impacts on *Ae. aegypti* and dengue are mediated by the aquatic container habitats, which host the immature stages.

The hourly development rate equation is Focks et al. (1993)'s implementation of the Sharpe and DeMichele (1977) model, which models *Ae. aegypti* development as a process driven by a temperature‐dependent rate‐controlling enzyme. Development rate is attenuated at high and low water temperatures due to (reversible) denaturing of this enzyme. The specific parameter values we use in this equation are identical to those used in CIMSiM and Skeeter Buster (Table S4 in Supporting Information S1). Development rate contributes to cumulative development (the product of development rate and time), which is what directly impacts *Ae. aegypti* growth. An *Ae. aegypti* individual must exceed a threshold of cumulative development of 0.95 before they transition from one life stage to the next (e.g., an egg hatching into a larva).

Daily survival rate is computed as the product of a base daily survival rate (0.99) and two survival factors: one associated with mortality due to water temperature extremes and the other associated with mortality due to desiccation. We briefly describe these components here; details can be found in (Text S3.2 in Supporting Information S1).

To determine the temperature‐based survival factor, we take the maximum and minimum water temperatures for a given day and apply them to the survival factor curve (Figure 3), which yields one survival factor based on the maximum temperature and one survival factor based on the minimum temperature. The product of these maximum and minimum temperature survival factors is the overall temperature‐based survival factor.

The desiccation‐based survival factor is only relevant if the container is dry: in a water‐filled container this survival factor is simply 1.00. In a dry container this survival factor is a flat value for larvae (0.05; very susceptible to desiccation) and pupae (0.95; resilient to desiccation), while for eggs the survival factor mildly depends on vapor pressure deficit (ranging from 0.95 to 0.99).

The daily survival rate accounts for mortality due to temperature extremes and desiccation, but there are other sources of mortality that we choose not to incorporate into our model given a lack of data that precisely captures their effects. Namely, in modeling daily survival rate we assume that the container habitats are not food‐limited (i.e., no mortality due to starvation) and that there is no predation (which Focks et al. (1993) notes as a commonly observed source of egg loss both in the laboratory and in the field).

One key limitation of the simulated water temperature data yielded by the WHATCH’EM model—which we use here in Step 2 to calculate the development and survival rates—is that WHATCH’EM is unable to calculate water temperatures when the water height is too low (<15 mm) due to numerical stability reasons. If left unaddressed, this would mean that we would be unable to calculate development and survival rates in low water height conditions. To address this issue, we estimate and fill in the missing water temperature values based on linear regressions on the MERRA‐2 2‐m air temperature data (for more details, see Text S2.1 in Supporting Information S1). The resulting uninterrupted water temperature data is what we use to calculate the development and survival rates.

While these models of vector biology are tremendously useful for gaining insight into the complexities of mosquito survival and development, we should also be thoughtful about the limitations of such models. In particular, modeling temperature‐based survival factor is challenging since laboratory‐based experiments cannot truly simulate a mosquito's exposure to temperature. In a laboratory, a mosquito usually experiences experiments where temperature is held constant or has a diurnal fluctuation that is identical from day‐to‐day (e.g., Mordecai et al., 2019, and references therein). However, in their natural environment, a mosquito experiences varying diurnal fluctuations of temperature as well as changes in mean temperature from day‐to‐day. Our modeling does not account for this complexity. We assume a mosquito's survival is solely based on the given day's temperature, with no memory of the mosquito's history of temperature experiences and how that might have a compounding influence on their survival (e.g., one might expect a mosquito who has barely survived extreme temperatures to be weaker and more likely to die in the future, but our modeling does not account for this).

Another facet of estimating temperature‐based survival factor is the choice of data/model (for extensive detail on this we refer readers to the compilation of mosquito thermal biology data in Mordecai et al., 2019). To consider our work's sensitivity to model choice we have tested our modeling pipeline with two models of temperature‐based survival factor. In this paper we primarily discuss our findings based on the temperature‐based survival model used by Focks et al. (1993) (the left inset plot in Figure 3). The second model we tested is one we have constructed based on data from Eisen et al. (2014). Testing of the second model is described in detail in (Text S6 in Supporting Information S1), but in brief: the results are generally similar to what we get using the Focks et al. (1993) model, suggesting that our central findings in this work are robust. We do find some differences in survival rate based on location and some differences in seasonal dynamics of the model outputs, so we believe that more deeply exploring our modeling pipeline's sensitivity to the choice of temperature‐dependent survival model would be a valuable path for future work.

#### 2.2.4 Step 3—Vector Population Dynamics

We simulate *Ae aegypti* population dynamics using a custom model based on the models CIMSiM and Skeeter Buster. Our model assesses the cumulative impact of the development and survival rates (from step 2) on a population of eggs, larvae, and/or pupae, tracking each individual's daily development, life stage transitions, and deaths over 1 month. If an individual develops and survives into adulthood, we consider that a “success” and cease tracking them (i.e., we do not track development and death for adults, as those do not directly depend on the aquatic container habitats that this work focuses on). Further details of the model are described in (Text S4.1 in Supporting Information S1). The model yields population sizes of each life stage for each day, but in our analysis we typically summarize the model results using one metric: the population size of adults at the end of month. These population sizes are *not* meant to realistically represent the number of individuals in a container; our interest is in *relative* differences in population sizes as a tool for assessing differing population growth under different climate/habitat conditions.

An important model consideration is the initial *Ae. aegypti* population (i.e., an initial population size, life stage, and starting day of month), which is specified by the user. Choosing these initial conditions requires some care so as to minimize the sensitivity of our results to various incidental sources of variability; after all, we are interested in variability in population dynamics that arises from climatic factors, not from other sources.

One such source of variability: the model has stochastic elements that introduce variance to the population dynamics, meaning that identical initial conditions will produce different results from one model run to the next. We mitigate this stochastic variance by choosing a large initial population of 1,000 individuals. We deemed this sufficiently large based on testing as described in (Text S4.2 in Supporting Information S1).

Another source of variability: the population dynamics may be sensitive to the day of month on which we introduce the initial population. For example, if we introduce the initial population on a day of the month that is particularly unfavorable for survival, that may yield a lower final population than if we had introduced the initial population a day later. Alternatively, if we introduce the initial population too late in the month, then they may simply not have enough time to develop into adults before the end of the month, regardless of their development and survival rates. We account for these by running the model multiple times; each of these runs corresponds to a different day of month on which the initial population is introduced, from the first to seventh days of the month.

A third source of variability: the initial life stage of the individual will influence the population dynamics, as each stage has different development/survival rates and later stages require fewer days of development and survival before reaching adulthood. Since a real container habitat may have all three immature stages coexisting at any given time, we echo this in our modeling by having an initial population of each immature stage: eggs, larvae, and pupae. We track these three populations separately (e.g., we separately count adults that were originally eggs, adults that were originally larvae, and adults that were originally pupae).

In summary, for each set of month‐long timeseries of development and survival rates from step 2, we simulate population dynamics given an initial population consisting of 1,000 individuals each of eggs, larvae, and pupae introduced on each of the first seven days of the month (a total of 21,000 individuals).

## 3 Results and Discussion

### 3.1 How Does the Model Translate Different Climate Conditions to Different Adult Populations?

Let us first assess whether the outputs produced by our modeling pipeline satisfy a basic requirement to have value as climate‐informed dengue predictors: does year‐to‐year variability in the input climate conditions systematically yield different output adult populations? For example, we might expect cool and wet years to yield adult populations that are comparable to one another, but differ from the simulated adult populations for hot and dry years. Having such model behavior is critical for the model outputs to have value in a climate‐informed dengue prediction system, as the model outputs cannot possibly predict the interannual variability in dengue incidence unless they themselves vary systematically from year to year.

**Figure 4:**
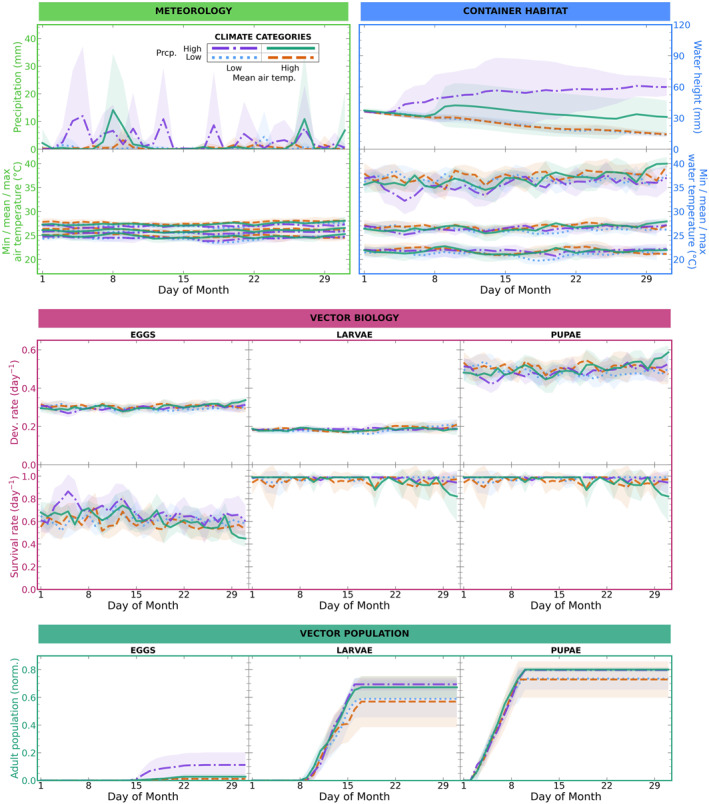
Timeseries of modeling pipeline input/output variables from all **January model runs for Jaffna** (2001–2020) for different climate categories. All variables are daily values. The variables are as follows: (meteorology) precipitation, minimum/mean/maximum 2‐m air temperature; (container habitat dynamics) water height, minimum/mean/maximum water temperature; (vector biology) development and survival rate for eggs, larvae, pupae; (vector population dynamics) population of adults from eggs, larvae, pupae, normalized to initial population. Each of the four climate categories represents a subset of the 20 years of data, sorted according to combinations of lower/upper quantiles of monthly mean 2‐m air temperature and lower/upper quantiles of monthly total precipitation. In each plot, each timeseries and its shading represents the mean and standard deviation across the years of data within a climate category: low air temperature + low precipitation (dotted blue line), high air temperature + low precipitation (dashed orange line), low air temperature + high precipitation (dash‐dotted purple line), high air temperature + high precipitation (solid green line).

We begin with Figure 4, an example highlighting the interannual variability of the model inputs/outputs and the step‐by‐step propagation of meteorological signals through the modeling pipeline. Figure 4 shows timeseries of model inputs/outputs from the January model runs for Jaffna, beginning with inputs of precipitation and 2‐m air temperature and ending with outputs of simulated adult populations from eggs, larvae, and pupae. Note that we omit showing the other meteorological inputs—relative humidity, soil temperature, and cloud cover—because they do not offer much additional information beyond what precipitation and air temperature already provide; they either have much less of an impact on the modeling pipeline than precipitation and air temperature do or (in the case of soil temperature) their behavior is similar to that of air temperature. We have focused this example on Jaffna in January because this is typically a time and location of high dengue indigence. For analogous figures for months of high dengue incidence in the other two locations—Negombo and Nuwara Eliya—please see (Figures S16 and S17 in Supporting Information S1).

In Figure 4, rather than showing the timeseries of every individual year of data (2001–2020), we instead show a timeseries for each of four climate categories. These climate categories represent distinct subsets of the 20 years of data, sorted according to combinations of lower/upper quantiles of monthly mean 2‐m air temperature and lower/upper quantiles of monthly total precipitation. This yields our four categories of low air temperature and low precipitation (hereafter referred to as *low temp/low prcp*), high air temperature and low precipitation (*HIGH TEMP/low prcp*), low air temperature and high precipitation (*low temp/HIGH PRCP*), and high air temperature and high precipitation (*HIGH TEMP/HIGH PRCP*). By examining model behavior across these climate categories, we assess the extent to which differences in input climate conditions due to interannual variability yield differences in simulated adult population outputs.

The outputs of the modeling pipeline show that there *are* generally differences in output based on the initial climate conditions: end‐of‐month adult populations are relatively high for the *low temp/HIGH PRCP* and *HIGH TEMP/HIGH PRCP* categories, but are relatively low for the *HIGH TEMP/low prcp* and *low temp/low prcp* categories. Moreover, within each pair of categories with similar precipitation (e.g., *low temp/HIGH PRCP* and *HIGH TEMP/HIGH PRCP*), the category with lower temperature tends to have higher end‐of‐month adult populations (e.g., *low temp/HIGH PRCP*). While there is some variance in these results (as indicated by overlapping 1‐standard deviation shading in Figure 4), intuitively these pattern makes sense. Jaffna is a hotter and drier location in Sri Lanka, so we might expect years with higher‐than‐normal precipitation and lower‐than‐normal air temperature to counteract the prevailing climate and yield aquatic habitats more suitable for the mosquito immatures. This intuition is a helpful starting point for considering climate‐mosquito population associations—and would be the limit of our understanding if our model was purely statistical—but with this mechanistic model we can delve deeper in our analysis: *why* does the model yield higher adult mosquito populations in years of higher‐than‐normal precipitation and lower‐than‐normal air temperature? To answer this question, we next consider the intermediate model outputs (Figure 4).

Why higher‐than‐normal precipitation would produce more favorable habitats is especially illuminating, as there are multiple potential mechanistic pathways at play. The first, perhaps more intuitive pathway, is that higher precipitation reduces the frequency with which the habitat dries out and becomes inhospitable for mosquito immatures. Yet, when we look at modeled water height, there are no model runs in which the container completely dries out. Therefore this mechanistic pathway does not explain the association we observe between precipitation and simulated adult population. The second mechanistic pathway is more nuanced: higher precipitation increases the water volume in container habitats, increasing the habitat's heat capacity and buffering it against dangerously high fluctuations in temperature. We do see evidence of this pathway in our model results. For example, compare the *low temp/HIGH PRCP* category (dash‐dotted purple line) to the *low temp/low prcp* category (dotted blue line). The *low temp/HIGH PRCP* category tends to have higher water height than *low temp/low prcp*. For both climate categories the habitat regularly reaches inhospitable temperatures, with the range of maximum daily water temperatures (∼30–40°C) falling squarely within the range of diminishing survival for mosquito immatures (>30°C for eggs and >39°C for larvae and pupae, per Figure 3). However, the *low temp/HIGH PRCP* category's maximum water temperature tends to be lower, which is more favorable for survival of the mosquito immatures. This translates to typically higher egg/larva/pupa survival rates—and therefore higher adult populations—for *low temp/HIGH PRCP* than for *low temp/low prcp.*

Why lower‐than‐normal temperatures would produce more favorable habitats is relatively straightforward: lower air temperature yields lower water temperature and, in a hotter location like Jaffna, this means a lower frequency of dangerously high water temperatures. As an example of this, compare the *low temp/low prcp* category (dotted blue line) to the *HIGH TEMP/low prcp* category (dashed orange line). For both categories the water height is comparable, so we expect any differences between these categories to be not because of the (precipitation‐driven) water temperature buffering effect discussed previously, but because of differences in air temperature. The *low temp/low prcp* category typically has a slightly lower maximum water temperature than the *HIGH TEMP/low prcp* category, in accordance with what we'd expect based on air temperature, and this translates to typically higher survival rates—and therefore higher adult populations—for *low temp/low prcp* than for *HIGH TEMP/low prcp.*

**Figure 5:**
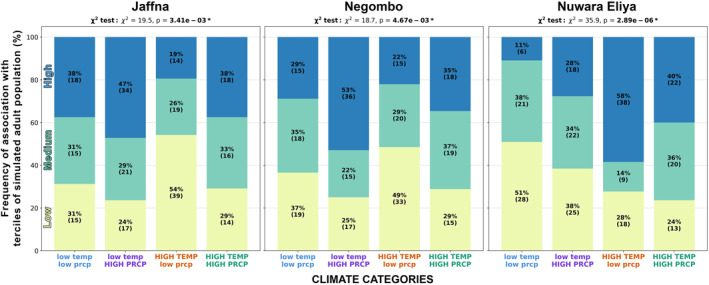
The associations between categories of monthly climate conditions and simulated monthly adult population from pupae. The four categories of climate conditions are combinations of lower/upper quantiles of monthly mean 2‐m air temperature and lower/upper quantiles of monthly total precipitation. The three categories of adult population from pupae (shown as the differently colored bars) are low, medium, and high terciles of population at end of month. Annotations within each bar indicate the amount of monthly adult population data points both as a percentage of all data points within that climate category and as a count of data points. The chi‐square test result shows a bolded p‐value if *p* < 0.05 (i.e., when we consider that the two categorical variables, climate categories and simulated adult population terciles, are not independent). These plots show data aggregated from all months of the year, but the categorization was done separately for each calendar month to minimize the impact of seasonality.

This example of the modeling pipeline shows how interannual variability in the input climate conditions (namely air temperature and precipitation) translates systematically to interannual variability in the simulated adult populations. However the evidence from Figure 4 alone is limited, as it just shows data for Jaffna in January. To systematically examine our model results for all locations and months we next take a higher‐level view, as seen in Figure 5. Here we show how the same four categories of input climate conditions are associated with categories of simulated adult population from pupae (terciles of low, medium, and high population at end of month) for each of our three locations of interest: Jaffna, Negombo, and Nuwara Eliya. All months of data are included here, but we sorted the data into climate categories and population terciles separately for each calendar month to minimize the impact of seasonality (e.g., for each calendar month, the 20 data points of adult population at end of month were labeled as low, medium, and high terciles; then the data for all calendar months were aggregated). We focus here on adult population from pupae rather than adult population from larvae or eggs as the results are broadly comparable, but one can see the results for adult population from larvae and from eggs in (Figures S18 and S19 in Supporting Information S1) (the major difference being that Nuwara Eliya's adult population from eggs is almost always zero for all climate categories).

There is a clear correspondence between the modeling pipeline's input climate conditions and the output simulated adult population from pupae. Some climate conditions are notably more favorable than others, and this association depends on location. For Jaffna and Negombo, *low temp/HIGH PRCP* tends to yield higher adult populations from pupae, while *HIGH TEMP/low prcp* tends to yield lower adult populations from pupae. For Nuwara Eliya, both *HIGH TEMP/low prcp* and *HIGH TEMP/HIGH PRCP* tend to yield higher adult populations from pupae, while *low temp/low prcp* tends to yield lower adult populations from pupae.

These results broadly match what we expect of these locations. Nuwara Eliya is a much cooler location in Sri Lanka, so higher‐than‐normal air temperatures would help produce tolerable habitat temperatures for the pupae. The amount of precipitation seems to play a secondary role here. Jaffna and Negombo are generally warmer locations in Sri Lanka, so having lower‐than‐normal air temperature presumably helps the habitat temperature for the pupae (i.e., the container water temperature) stay within the temperature range of maximum survivability. It is interesting that high precipitation is favorable for adult populations in both locations because in terms of precipitation Jaffna and Negombo are quite different: Jaffna is dry, Negombo is wet. We might have expected a wetter location like Negombo to instead have lower adult populations in high precipitation conditions, as too much precipitation can suppress adult populations by washing out immature stages from their container habitats (Koenraadt & Harrington, 2008). However we don't see this happening in our model results because of the container size and initial fill that we use; it doesn't typically rain enough within a month to fill and then overflow the modeled container. Thus the high precipitation is solely beneficial for the immature individuals: more water volume in the container habitat means that daily water temperature fluctuations are attenuated, mitigating mortality due to excessively high or low temperatures. Over a longer simulation period, or for simulations with different initial conditions, these results might be different.

It is evident that the model's input climate conditions have an impact on the output simulated adult populations, as demonstrated here with air temperature and precipitation. Interannual variability propagates through the model systematically. This is promising for the potential of the model outputs to serve as dengue predictors, inasmuch as the climate sensitivities of the mosquito vector are what drive the interannual variability of dengue. We explore this notion in the subsequent sections by comparing our model outputs to observed dengue incidence.

### 3.2 What Seasonal and Interannual Variability Is There in the Model Variables?

**Figure 6:**
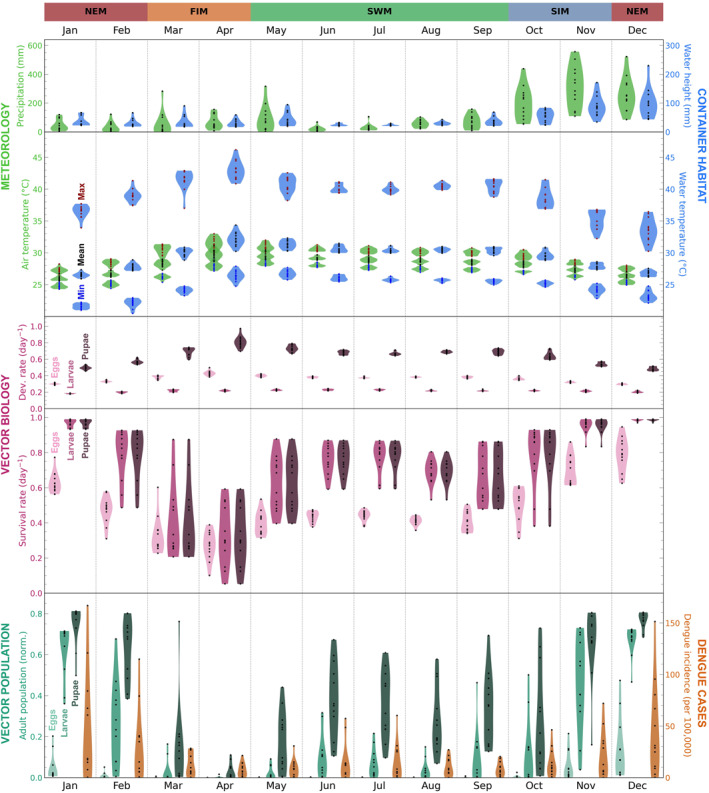
Violin plots of modeling pipeline input/output variables and recorded dengue incidence for **Jaffna** (2007–2020, excluding outbreak years 2017 and 2019). All variables have been resampled to monthly values such that the violin plot for each calendar month contains one datapoint for each year of data. The variables are as follows: (meteorology) total precipitation, minimum/mean/maximum 2‐m air temperature; (container habitat dynamics) mean water height, minimum/mean/maximum water temperature; (vector biology) mean development and survival rate for eggs, larvae, pupae; (vector population dynamics) population of adults from eggs, larvae, pupae at end of month, normalized to initial population. At the top of the figure the months are labeled with Sri Lanka's four monsoonal seasons: NEM (northeast monsoon, December–February), FIM (first intermonsoon, March–April), SWM (southwest monsoon, May–September), SIM (second intermonsoon, October–November).

**Figure 7:**
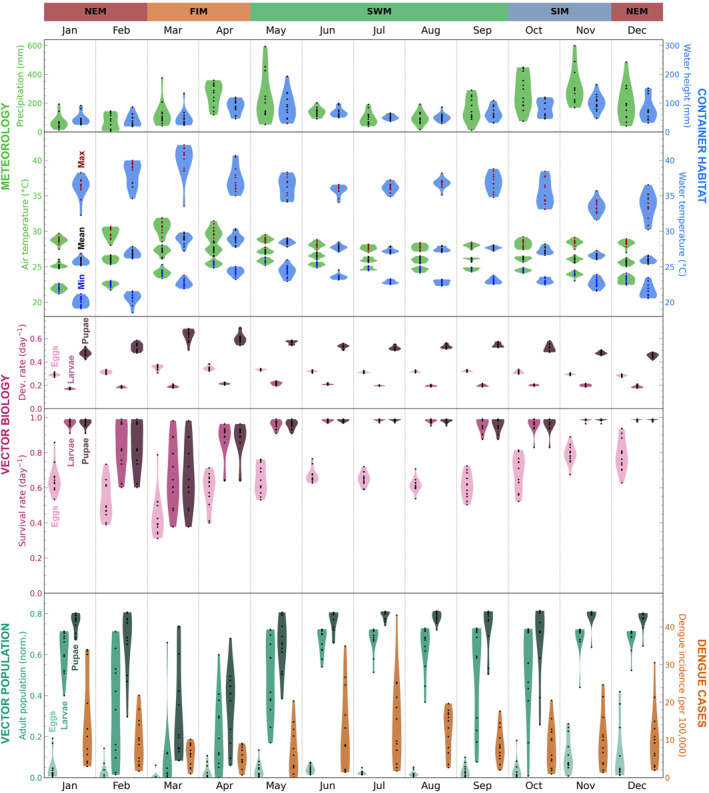
As in Figure 6, but for **Negombo**.

**Figure 8:**
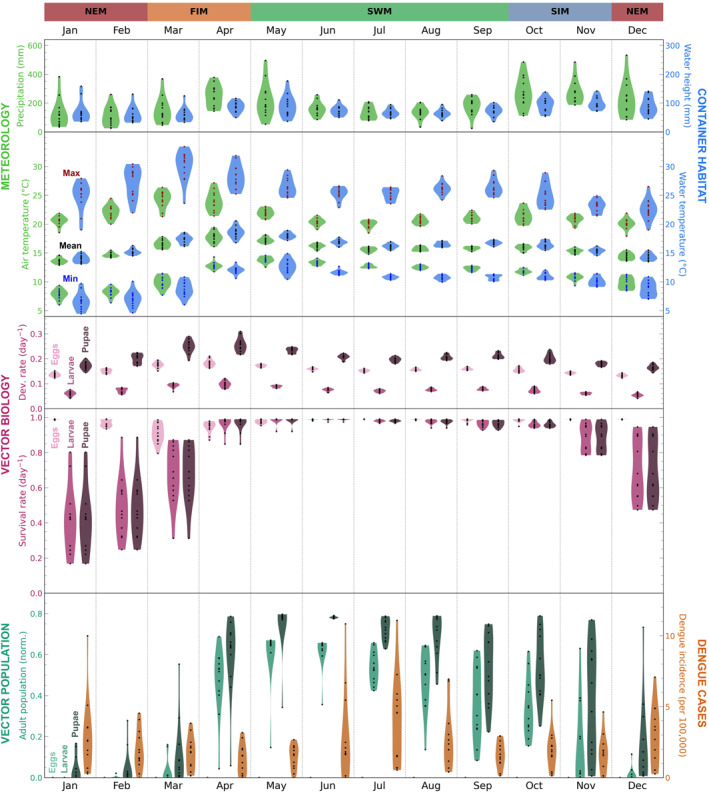
As in Figure 6, but for **Nuwara Eliya**.

Here we investigate the variability in the model variables in terms of both season‐to‐season and year‐to‐year differences and compare variability in observed dengue incidence. Here we only consider data within the time range of the dengue case data (2007–2020) and we exclude the years 2017 and 2019, when there were unique disease dynamics due to dengue outbreaks (associated with changes in dengue serotype prevalence and flooding events (Jampani & Amarnath, 2024; Malavige et al., 2021; Prabodanie et al., 2020; Tissera et al., 2020)). We consider the same model variables as in Figure 4, but resampled to monthly values: total precipitation; mean 2‐m air temperature; mean water height; mean water temperature; mean survival and development for eggs, larvae, pupae; and end‐of‐month population of adults from eggs, larvae, pupae. These variables are shown in Figure 6 (Jaffna), Figure 7 (Negombo), and Figure 8 (Nuwara Eliya), where the violin plots show interannual variability for each calendar month ((e.g., the January violin plot is based on the monthly data for all Januarys from 2007 to 2020, excluding 2017 and 2019). When discussing seasonal patterns we will refer to the four monsoon‐based season of Sri Lanka: northeast monsoon (December–February), first intermonsoon (March–April), southwest monsoon (May–September), second intermonsoon (October–November).

We note here that our comparison of model variables and observed dengue incidence is an optimistic exploration of potential associations between the two; we do not expect the two to neatly align. After all, the model variables solely represent climatic impacts on mosquito container habitat suitability and do not represent climatic impacts on *other* drivers of dengue (e.g., human mobility; adult mosquito life history traits such as lifespan, gonotrophic development, and biting rate), nor do they represent *non‐climatic* drivers of dengue (e.g., the dominant circulating dengue serotype in a location at any given time; the level of immunity the local people have to the dominant dengue serotype). We also expect a time lag between patterns in the model variables and patterns in observed dengue incidence. For example, we might expect dengue incidence to peak after mosquito population peaks, given that it takes time for mosquitoes to bite and infect people with dengue, for the disease to manifest symptoms, and for infected people to seek care and be recorded as dengue cases. So, we compare model variables to dengue incidence with the understanding that variability in the model variables may contribute to some, but certainly not all, of dengue dynamics.

#### 3.2.1 General Variability Patterns

While some patterns in the model variables are location‐specific (and will be discussed separately for each location in the following sections), there are numerous patterns that are common to all three locations (Figures 6, 7, 8; or, for ease of comparing across locations, see the compilation of June and December data in Figure S20 in Supporting Information S1). For one, the seasonality of the container habitat dynamics variables closely reflects meteorology, with water height qualitatively matching precipitation and water temperature qualitatively matching 2‐m air temperature. The development rates for eggs, larvae, and pupae qualitatively have the same seasonal patterns as water temperature. Survival rates for eggs, larvae, and pupae are generally lower when development rates are higher. This is because survival rate decreases with increasing maximum daily water temperature while development rate generally increases with increasing water temperature (with one exception: larval development rate decreases as temperatures increase past ∼31°C) (recall Figure 3). In months that are extremely favorable for survival the daily survival rates can be near their maximum possible value (0.99) in every year, yielding minimal interannual variability (e.g., Jaffna larvae/pupae in December; Figure 6 or Figure S20 in Supporting Information S1).

The end‐of‐month adult populations from initial stages of eggs, larvae, and pupae generally have greater interannual variability than the development and survival rates. This is understandable, since simulating *Ae. aegypti* individuals' development and survival over a month to calculate population size has a compounding effect. However there are certain calendar months when the interannual variability in adult population sizes is minimal, either because conditions are highly unfavorable and the population is near zero every year (e.g., Jaffna eggs in June; Figure 8 or Figure S20 in Supporting Information S1) or because conditions are highly favorable and the population is near its maximum possible value every year (e.g., Nuwara Eliya pupae in June; Figure 8 or Figure S20 in Supporting Information S1). In considering the adult populations we also find that, if the initial stage is more mature, the resulting adult population is generally higher (e.g., the adult population from pupae is generally greater than the adult population from eggs). This is because individuals that are at a later stage of development at the start of the model run have less of a development/survival barrier to overcome to reach adulthood. Notably, the adult population from eggs is low (or even zero) for most calendar months, which suggests that egg development/hatching presents a large climatic barrier within the *Ae. aegypti* lifecycle.

In comparing the model variables with observed dengue incidence, we generally find that months of high end‐of‐month adult population sizes correspond to months of high dengue incidence. There is a similar, but weaker correspondence between high survival rate and high dengue incidence.

#### 3.2.2 Jaffna‐Specific Variability Patterns

Figure 6 shows violin plots for each model variable of interest for each calendar month, along with violin plots showing recorded dengue incidence for Jaffna for 2007–2020. In this section we note how Jaffna differs from Negombo and Nuwara Eliya, as the commonalities among all three locations have already been discussed in Section 3.2.1.

Jaffna is a relatively hot and dry city in Sri Lanka, as seen in the violin plots for rainfall and 2‐m air temperature. Heavy rainfall is focused in the second intermonsoon season and early northeast monsoon season and varies substantially from year to year. Air temperature peaks in April and May, when the first intermonsoon season transitions into the southwest monsoon season. The container maximum water temperature values are dramatically higher than air maximum temperature values (by about 5–10°C) and are high enough to threaten mosquito survival. Survival rates for eggs, larvae, and pupae appear almost like the inverse of the development rates, and the survival rates for larvae and pupae differ from the survival rates for eggs: they have greater interannual variability because the larval and pupal temperature‐dependent survival factors are more sensitive to temperature fluctuations, changing more dramatically for a given change in water temperature (recall Figure 3). The end‐of‐month adult populations from eggs, larvae, and pupae all generally peak from November through January as the second intermonsoon season transitions into the northeast monsoon season. Adult populations are particularly low in the first intermonsoon season, when high temperatures lead to low survival rates.

In comparing the adult populations to dengue incidence, there is potential to link the observed dengue seasonality signal to the simulated adult populations (and particularly the adult population from pupae): both have a primary peak in the northeast monsoon season and a secondary peak in the southwest monsoon season.

#### 3.2.3 Negombo‐Specific Variability Patterns

Figure 7 shows violin plots for each model variable of interest for each calendar month, along with violin plots showing recorded dengue incidence for Negombo for 2007–2020. In this section we note how Negombo differs from Jaffna and Nuwara Eliya, as the commonalities among all three locations have already been discussed in Section 3.2.1.

Negombo is a relatively warm and wet city in Sri Lanka. Rainfall shows two large seasonal peaks, one at the end of the first intermonsoon season and another in the second intermonsoon season. Air temperature peaks in the first intermonsoon season and is slightly lower than for Jaffna (by about 1–3°C). The container maximum water temperature values are far higher than air maximum temperature values (by about 5–10°C, as in Jaffna) and reach temperatures that compromise mosquito survival. The larval development rates differ from the egg and pupal development rates, peaking later in the year in May rather than in March. This is because, while development rate typically increases with increasing water temperature, larval development rate falls when temperatures are as high as they are in March (recall Figure 3). Survival rates for larvae and pupae are quite different from survival rates for eggs; for most of the year they are nearly their maximum possible value of 0.99 (i.e., 99% survival). However, survival rates decrease dramatically in the first intermonsoon season because the maximum daily temperatures are extreme enough to cause temperature‐related mortality.

The end‐of‐month adult populations from eggs are quite different from the adult populations from larvae and pupae, echoing the differences in survival rate. Adult population from eggs is low for most of the year, but peaks at the end of the second intermonsoon season. In contrast, adult populations from larvae and pupae are high during the middle of the southwest monsoon season (∼July) and as the second intermonsoon season transitions into the northeast monsoon season (November and December). Adult populations from larvae and pupae drop steeply in the first intermonsoon season, reflecting the lower survival rates during this time. For some months the adult populations show extreme interannual variability, with near zero population in some years but near maximum in other years (e.g., larvae in February and September; pupae in March and April).

The adult populations capture some of the seasonal cycle of dengue incidence, showing two annual peaks. One is in the southwest monsoon season and the other is in the northeast monsoon season, but the latter peak is earlier than for dengue incidence (November/December rather than January). The seasonal patterns in adult populations are obfuscated, though, by extreme values and high interannual variability; in most months there is near‐zero adult population from eggs, in peak months there is near‐maximum adult population from larvae/pupae, and in some months the adult populations span the entire range of possible population sizes (e.g., adult populations from larvae in February). The adult populations do not capture the relative sizes of the dengue peaks. In fact, the adult population from eggs shows the opposite, with a larger peak in the northeast monsoon season rather than in the southwest monsoon season. So, while we do see two peaks in the adult populations as we do for dengue incidence, the adult populations do not clearly convey the finer details of dengue seasonality.

#### 3.2.4 Nuwara Eliya‐Specific Variability Patterns

Figure 8 shows violin plots for each model variable of interest for each calendar month, along with violin plots showing recorded dengue incidence for Nuwara Eliya for 2007–2020. In this section we note how Nuwara Eliya differs from Jaffna and Negombo, as the commonalities among all three locations have already been discussed in Section 3.2.1.

The mountainous city of Nuwara Eliya is relatively cold and wet. Seasonal patterns of precipitation and air temperature are qualitatively similar to those for Negombo, with precipitation peaking at the end of the first intermonsoon and in the second intermonsoon, while air temperature peaks in the first intermonsoon. However, Nuwara Eliya is much colder than both Negombo and Jaffna (by ∼10°C in any given month) and in a temperature regime where mosquito survival will likely be determined by the container minimum temperatures (rather than the maximum temperatures). Container minimum water temperature is slightly lower than air minimum temperature (by about 1–2°C). Survival rates for eggs, larvae, and pupae are maximal (0.99) in most months, with notable exceptions: the survival rate for eggs drops dramatically (albeit with high interannual variability) in the first intermonsoon season, while the survival rate for larvae and pupae has a minimum in the northeast monsoon season. These troughs in survival rate are driven by different extremes in water temperature: the decrease in egg survival rate is due to harmful maximum temperatures, while the decrease in larval/pupal survival rate is due to harmful minimum temperatures. Eggs are affected differently than larvae/pupae because they have different temperature thresholds for survival rate (recall Figure 3).

The end‐of‐month adult populations from eggs is zero for almost every model run. This is because eggs can only hatch in the model if they are submerged in water that is at least 22°C, a temperature that the water almost never reaches in these model runs. Adult populations from larvae and pupae show one annual peak, with a rapid rise in the first intermonsoon season, near‐maximum population at the beginning of the southwest monsoon season, a precipitous drop in the second intermonsoon season, and minimal population in the northeast monsoon season. This generally reflects the seasonal patterns in survival rate for larvae and pupae.

The adult populations from eggs lend no insight into dengue incidence since they are almost always zero, but the adult populations from larvae and pupae partially capture dengue seasonality. The adult populations from larvae and pupae have a peak in the southwest monsoon season, a little earlier than the primary peak in dengue incidence. However, there is no secondary peak in the northeast monsoon season; at this time the adult populations are close to zero. Thus we find that only part of the dengue seasonality signal (one of two peaks) is captured in the adult populations for Nuwara Eliya. Given that Nuwara Eliya appears so climatically unfavorable for dengue in the northeast monsoon season, perhaps the dengue peak at this time is driven by non‐climate factors (e.g., human travel to/from other cities that do have a peak in dengue incidence during the northeast monsoon season).

### 3.3 How Closely Associated Is the Interannual Variability in the Model Variables With the Interannual Variability in Dengue Incidence?

We now assess associations between the model variables and recorded dengue incidence. This assessment aims to identify the extent to which the model variables might explain some (but not all) of the variability in dengue incidence; as detailed in Section 3.2, there are many factors that influence dengue incidence that the model does not account for. We are interested in answering two questions. First, are there strong associations between model variables and dengue incidence that might indicate predictive skill? Second, are the associations between model variables and dengue incidence at least as strong as those between meteorological variables and dengue incidence? After all, existing dengue early warning systems already take advantage of associations between meteorological variables and dengue incidence, and our hope here is that the outputs of the modeling pipeline can at least match (and ideally surpass) these associations.

**Figure 9:**
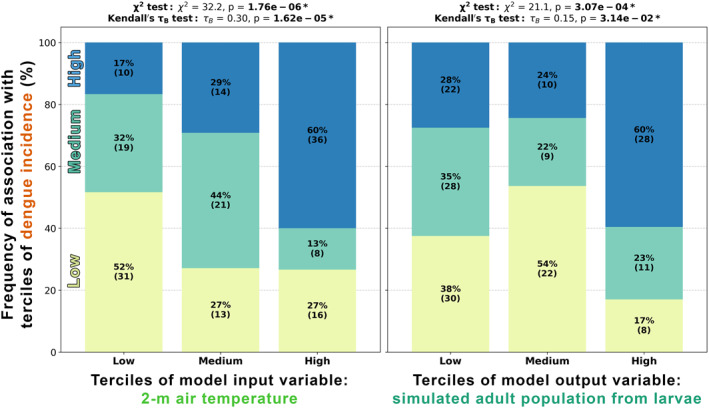
The associations of (left) terciles of monthly mean 2‐m air temperature (lagged by two months) and (right) terciles of simulated end‐of‐month adult population from larvae (lagged by two months) with terciles of monthly dengue incidence for **Nuwara Eliya** (2007–2020). Annotations within each bar indicate the amount of data points both as a percentage of all data points contributing to that category and as a count of data points. Statistical results for the chi‐square test and Kendall's tau‐b test show a bolded p‐value if *p* < 0.05. These plots show data aggregated from all months of the year, but the categorization was done separately for each calendar month to minimize the impact of seasonality.

To address these questions, we compare terciles of each model variable against terciles of dengue incidence, as in Figure 9. Similar to Figure 5, we minimized impacts of seasonality by converting all variables from daily to monthly values and by computing terciles for each calendar month before aggregating the results. The model variables included in this analysis were: mean 2‐m air temperature; mean of daily maxima of 2‐m air temperature; mean of daily minima of 2‐m air temperature; total precipitation; mean container water temperature; mean of daily maxima of container water temperature; mean of daily minima of container water temperature; mean container water height; mean survival rate for eggs, larvae, pupae; mean development rate for eggs, larvae, pupae; and end‐of‐month adult population from eggs, larvae, pupae. We find that the most notable associations with dengue incidence are for Nuwara Eliya adult populations from larvae when considering all years of data (including outbreak years) and with lags of 1 and 2 months (Figure 9 shows just the associations with 2‐month lag). These scenarios have a statistically significant chi‐square test result indicating a lack of independence between the two variables (adult populations from larvae and dengue incidence) as well as a statistically significant Kendall's tau‐b test result indicating a directionality to the relationship (higher adult populations from larvae are associated with higher dengue incidence). In these scenarios, a high tercile value of simulated adult population from larvae often corresponds to a high tercile value of dengue incidence (∼60% of the time in our timespan of data).

We also find statistically significant, but weaker associations with dengue incidence in other scenarios for Nuwara Eliya adult population, as well as for the model variables of survival rate, development rate, container water temperature, and minimum/mean/maximum mean air temperature (primarily for Nuwara Eliya). So we find that in some model scenarios, and particularly for Nuwara Eliya, there are notable associations between model variables and dengue incidence. Importantly, these dengue associations are not consistent across locations; while meteorological relationships with dengue incidence may explain disease dynamics in one location, these relationships do not necessarily directly translate to other locations (or even to the same location under a changing climate). This highlights the value of location‐specific mechanistic modeling for robustly assessing how climate impacts dengue incidence.

**Figure 10:**
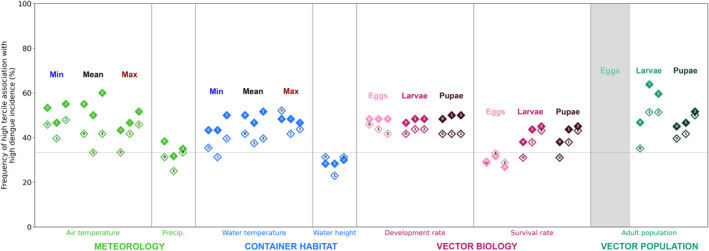
The frequency of association between the high tercile of dengue incidence and the high tercile of each modeling pipeline input/output variable for **Nuwara Eliya** (2007–2020). Note how this figure and Figure 9 are related: the high tercile‐high tercile percentage from each plot of Figure 9 corresponds to an individual data point in this figure. The modeling pipeline variables (all resampled to monthly values) are as follows: (meteorology) minimum/mean/maximum 2‐m air temperature, total precipitation; (container habitat dynamics) minimum/mean/maximum water temperature, mean water height; (vector biology) mean development and survival for eggs, larvae, pupae; (vector population dynamics) population of adults from eggs, larvae, pupae at end of month. For each modeling pipeline variable there are six data points corresponding to combinations of different lag times (0, 1, and 2 months; indicated by the number within the data point) and different years of data (including or excluding the outbreak years of 2017 and 2019; indicated by a filled or not filled data point, respectively). The dashed line indicates the expected frequency of association if the associations among terciles were random (33%). For Nuwara Eliya the data for population of adults from eggs is omitted, as these values were mostly zero and therefore do not map clearly to terciles.

Now for the second question—are these associations are at least as strong as those between meteorological variables and dengue incidence? We find that precipitation does not have a statistically significant association with dengue incidence (at least within the framing of this analysis, where we split monthly total precipitation into terciles). Air temperature, however, does show statistically significant associations, and as an example we show, for Nuwara Eliya, a tercile plot for air temperature alongside the tercile plot for adult populations from larvae, where we previously showed strong associations with dengue incidence (Figure 9). We find that the results for air temperature and for adult populations from larvae are fairly comparable for Nuwara Eliya. There are some minor differences (e.g., low air temperature is more often associated with low dengue incidence than low adult population from larvae is), but nothing so drastic as to clearly indicate a stronger dengue association for one variable over the other. This is also seen when comparing the frequency of high tercile‐high tercile associations with dengue incidence across all the modeling pipeline variables (Figure 10): air temperature and adult population from larvae stand out as having the highest frequency of high tercile‐high tercile association with dengue incidence. In contrast, for Jaffna and Negombo, the variables that have the highest frequency of high tercile‐high tercile association with dengue incidence are air temperature, water temperature, and development rates (Figures S21 and S22 in Supporting Information S1).

These findings suggest that the dengue associations of some model variables (adult population from larvae for Nuwara Eliya; water temperature and development rates for Jaffna and Negombo) are comparable in strength to the dengue associations of air temperature. From the perspective of statistical forecast development, then, it is not clear whether our simulations are necessary; one might do just as well using meteorology as a direct predictor of dengue incidence. But in providing a mechanistic link between meteorology and a hypothesized driver of dengue risk (i.e., *Ae. aegypti* habitat suitability and population size), the modeling pipeline offers process‐based explanation. If the simulations had indicated that anomalies in air temperature did not propagate through to create differences in habitat conditions or mosquito survival, then we would be left to wonder how and why the climate signal influences dengue incidence. The modeling pipeline also provides a tool for simulating interactions between climate variability and *Ae. aegypti* control measures, such as emptying containers.

## 4 Conclusions

Using our mechanistic modeling pipeline, simulated *Ae. aegypti* population sizes show associations with observed dengue incidence that are comparable in strength to the associations between 2‐m air temperature and observed dengue incidence. The simulated population sizes also generally show seasonal cycles similar to those of dengue. Notably, for Nuwara Eliya, simulated population sizes that are higher than normal for a given month usually correspond to higher‐than‐normal dengue incidence (∼60% of the time). This suggests that the interannual variability of the simulated population sizes, inherited as it is from the interannual variability of the meteorological data and transformed by the mechanistic models, may contain some predictive skill of the year‐to‐year variations in dengue incidence.

These findings must be thoughtfully considered alongside the limitations of our modeling approach. First, our findings avoid making any strong conclusions about climate‐dengue associations in dengue outbreak years, even though outbreak years would be useful to predict in support of public health interventions. We omit this because there are only two dengue outbreak years in our data set (2017 and 2019), making it difficult to assert with statistical confidence that outbreak years are associated with particular climatic anomalies. An analysis of outbreak years is worth pursuing in the future with this model, perhaps in combination with data on dengue serotype prevalence and flooding events, both of which have been associated with the 2017 and 2019 outbreaks (Jampani & Amarnath, 2024; Malavige et al., 2021; Prabodanie et al., 2020; Tissera et al., 2020). Second, we recognize that most dengue reporting occurs in the large tertiary care centers where the diagnosis is made, so cases may be diagnosed and reported from locations different from the patient's residence. This could introduce spatial misclassification, particularly in densely connected urban areas. Third, there may be diagnostics and surveillance heterogeneity, as diagnostic protocols and reporting practices may vary across health facilities. To overcome these limitations, further work using more granular, community‐level, or active surveillance data would improve model calibration and validation.

There are also numerous potential avenues of improvement for the modeling pipeline that may enhance associations between the model outputs and dengue incidence. These potential improvements include the use of more precise, location‐specific container specifications (e.g., based on entomological surveys of container breeding habitats), the incorporation of more recent models of *Ae. aegypti* environmental/thermal sensitivities (e.g., Mordecai et al., 2019), and the extension of the modeling pipeline to simulate the climatically driven life history and feeding behavior of *Ae. aegypti* adults (e.g., incorporating temperature‐dependent biting rate and adult lifespan (Mordecai et al., 2019)). Sustained entomological surveys of *Ae. aegypti* are also a priority, as we do not presently have the mosquito data required for direct evaluation of our simulated variability in mosquito populations.

That being said, there is also great practical utility in developing this modeling pipeline to function *without* relying on difficult‐to‐gather data such as local container distribution. Adapting this into a simplified, user‐friendly early warning system for dengue risk may particularly have value in resource‐constrained settings where detailed entomological or surveillance data may not be available. By leveraging the associations observed between meteorological parameters—such as temperature and rainfall patterns—and vector dynamics, a version of the model could be designed to accept routinely available meteorological inputs and generate risk alerts or guidance for preemptive vector control. Such a tool, deployed at the municipal or regional level, could support more proactive and cost‐effective public health responses. Future work will be needed to streamline model complexity, enhance accessibility through mobile or web‐based interfaces, and integrate with local public health infrastructure and early response protocols. In this vein, we encourage collaborations to integrate this modeling pipeline into an existing early warning system framework, testing the added value of container habitat modeling for prediction of dengue risk and evaluating the extent of data that is required for this model to produce robust and actionable results.

Our work thus far suggests that our process‐based modeling pipeline of breeding habitats may have value in predicting years of higher dengue risk—perhaps at least as well as predictions based on meteorology—and is worth further testing of its utility for dengue forecasting. The modeling pipeline's mechanistic structure offers greater understanding of the pathways by which climate impacts dengue dynamics and also presents opportunities to simulate the effectiveness of interventions (e.g., manual container emptying) and the changing landscape of dengue under future climate scenarios. Of course, an essential caveat to any climate‐dengue work is that the climatic lens on dengue that we present here is just one piece of a vast puzzle: dengue is a complex, ever‐evolving public health challenge that crosses disciplines and borders. Mitigating the harm that it does to people around the world will continue to be an exercise in communication, science, and policy, and, particularly, in recognizing the intricate web that connects humans, mosquitoes, and dengue to one another within our shared environment.

## Conflict of Interest

The authors declare no conflicts of interest relevant to this study.

## Supporting information

Supporting Information S1

## Data Availability

Further details on the modeling pipeline can be found in Supporting Information S1 and all code for modeling and data analysis can be found on GitHub (Yasanayake et al., 2025). This includes the modified version of WHATCH’EM used for this work, which differs from the original in (a) including calculation of vapor pressure deficit (a parameter relevant for vector mortality due to desiccation) and (b) having adjusted formatting of output files to better integrate into our modeling pipeline. The original WHATCH’EM model's documentation and source code can be found online (Steinhoff et al., 2016; Steinhoff & Monaghan, 2013). The MERRA‐2 and IMERG meteorological input data for our model are available online (Global Modeling and Assimilation Office, 2015a, 2015b, 2015c; Huffman et al., 2023), as are the Global Surface Summary of the Day weather station data used for bias correction (NOAA National Centers of Environmental Information, 1999). The Sri Lanka dengue case data and population data used to calculate dengue incidence are publicly available (Sri Lanka Ministry of Health – Epidemiology Unit, 2025; Central Bank of Sri Lanka, 2023; Sri Lanka Department of Census and Statistics, 2025). Data analysis and plotting were done using Python v3.9.16 (Python Software Foundation, 2022) with additional packages matplotlib v3.9.4 (Hunter, 2007), pandas v2.2.2 (The Pandas Development Team, 2024), camelot v0.11.0 (Camelot Developers, 2023), xarray v2024.7.0 (Hoyer & Hamman, 2017), numpy v1.26.4 (Harris et al., 2020), seaborn v0.13.2 (Waskom, 2021), scipy v1.13.1 (Virtanen et al., 2020), and scikit‐learn v1.6.1 (Pedregosa et al., 2011). The flowcharts in Text S4 (Figures S7–S9 in Supporting Information S1) were created in Canva. All other figures were created in Python with final formatting done using Adobe Photoshop.
