# Supplementary material for: Mechanistic Modeling of Aedes aegypti Mosquito Habitats for Climate‐Informed Dengue Forecasting
Source: Geohealth. 2025 Sep 16;9(9):e2025GH001376. doi: 10.1029/2025GH001376 (PMC12439285; doi:10.1029/2025GH001376)
Supplement: Supplementary file 1 — Supporting Information S1 [file GH2-9-e2025GH001376-s001.pdf]

GeoHealth

Supporting Information for

Mechanistic Modeling of *Aedes aegypti* Mosquito Habitats for Climate-informed Dengue Forecasting

C. N. Yasanayake[1], B. F. Zaitchik[1], A. Gnanadesikan[1], L. M. Gardner[2,3], and A. Shet[4]

[1]Johns Hopkins University Department of Earth & Planetary Sciences. [2]Johns Hopkins University Department of Civil & Systems Engineering. [3]Johns Hopkins University Department of Epidemiology. [4]Johns Hopkins University Department of International Health.

Contents of this file

Text S1 to S6
Figures S1 to S31
Tables S1 to S5

Introduction

The supporting information presented here expands upon the modeling pipeline described in the main text. In particular, we detail data processing steps and model structure/parameters that would be of interest to a reader focused on this work's methodology.

This document is structured to mirror the steps of the modeling pipeline, with all text, figures, and tables broadly organized under Text S1, S2, S3, S4, and S5. Text S1 discusses the meteorological and environmental inputs to the modeling pipeline and our method of bias correction for air and ground temperature data. Text S2 discusses the container habitat dynamics modeling using WHATCH'EM (Step 1 of the modeling pipeline), including relevant parameter values and our method of estimating missing water temperature values. Text S3 pertains to the vector biology modeling (Step 2), presenting the equations used to simulate mosquito development and survival. Text S4 concerns itself with the vector population dynamics modeling (Step 3), presenting the algorithms that underlie this modeling and discussing our efforts to minimize the variance in the model outputs (simulated adult population of *Ae. aegypti*) that arises from the model's inherent stochasticity. Text S5 contains assorted supporting figures referenced by the main text.

Text S1.
Modeling pipeline inputs – meteorology and environment

Here we discuss the meteorological and environmental data that serve as inputs into the modeling pipeline, detailing our method of bias correction (Text S1.1).

Text S1.1. Bias correction of meteorological data

The inputs to our modeling pipeline are air temperature, relative humidity, cloud cover, soil temperature, and vapor pressure deficit data from MERRA-2 (a global reanalysis data product) (Global Modeling and Assimilation Office, 2015a,b,c) and precipitation data from IMERG (a global data product that combines gauge observations and satellite data) (Huffman et al., 2023). Since these data products are not simply ground observations of meteorology they may have inherent biases, misrepresenting actual meteorological conditions to some degree. Therefore, before using these datasets for our work, we first assessed and mitigated these biases as described here.

We resampled the hourly MERRA-2 and IMERG meteorological data to daily values to match the cadence of available data from WMO-reporting weather stations (NOAA National Centers of Environmental Information, 1999) for each of the three locations (Negombo, Nuwara Eliya, Jaffna) and for the variables that had corresponding station data: daily total precipitation, daily mean/minimum/maximum air temperature, and daily mean relative humidity. We then compared the data distributions for MERRA-2/IMERG and the station data (Figure S1).

We find that the IMERG precipitation data agrees reasonably well with station data, so in this work we have used the IMERG data as is, without any bias correction. As for the MERRA-2 data, they agree sufficiently with stations data for Negombo and Jaffna that we do not bias correct for these locations (Figure S1; first and second rows). Among these data the most apparent discrepancy is for Negombo vapor pressure deficit, yet we do not expect this to be a major issue for our modeling pipeline, which has low sensitivity to such small differences in vapor pressure deficit (see Equation S10 and associated text).

For Nuwara Eliya, though, there is a substantial discrepancy between MERRA-2 data and station data: the MERRA-2 mean, minimum, and maximum daily temperatures appear to be about 6°C higher than the corresponding station data (Figure S1; third row). This temperature bias is problematic for our modeling pipeline, as these ~6°C shifts often cross the temperature thresholds for decreased survival (Table S5) and alter the modeled survival rates of the mosquito eggs, larvae, and pupae. We also found that the MERRA-2 ground temperature data for Nuwara Eliya was high enough to suggest a positive temperature bias. Therefore we bias-corrected the MERRA-2 air and ground temperatures for Nuwara Eliya to better match the station data.

We bias-corrected the Nuwara Eliya MERRA-2 air and ground temperature data using a linear regression against the station data. Bias-correcting MERRA-2 air temperature was relatively straightforward: since we have station data for air temperature, we created a linear fit of MERRA-2 air temperature to station air temperature and applied this linear relationship to each MERRA-2 air temperature data point. This shifted the MERRA-2 air temperature data points closer to the corresponding station data points (Figure S1; fourth row). Bias-correcting MERRA-2 ground temperature data was more nuanced: since we do not have ground-based soil temperature, we bias-corrected the ground temperature based on the same linear regression we used for air temperature—a linear regression of MERRA-2 air temperature on station air temperature. In other words, we assumed that the mathematical relationship

between MERRA-2 air temperature and station air temperature was the same as the relationship between MERRA-2 soil temperature and the actual soil temperature.

Our bias correction of Nuwara Eliya MERRA-2 temperature data is also relevant for other meteorological model inputs for Nuwara Eliya that are derived from air temperature: relative humidity and vapor pressure deficit. We computed relative humidity for Nuwara Eliya using the original air temperature data rather than the bias-corrected air temperature data to maintain consistency among the multiple MERRA variables used to calculate relative humidity (2-m air temperature, 2-m specific humidity, and surface pressure); using bias-corrected 2-m air temperature yielded unrealistic values of relative humidity. However we computed vapor pressure deficit for Nuwara Eliya using the bias-corrected air temperature data.

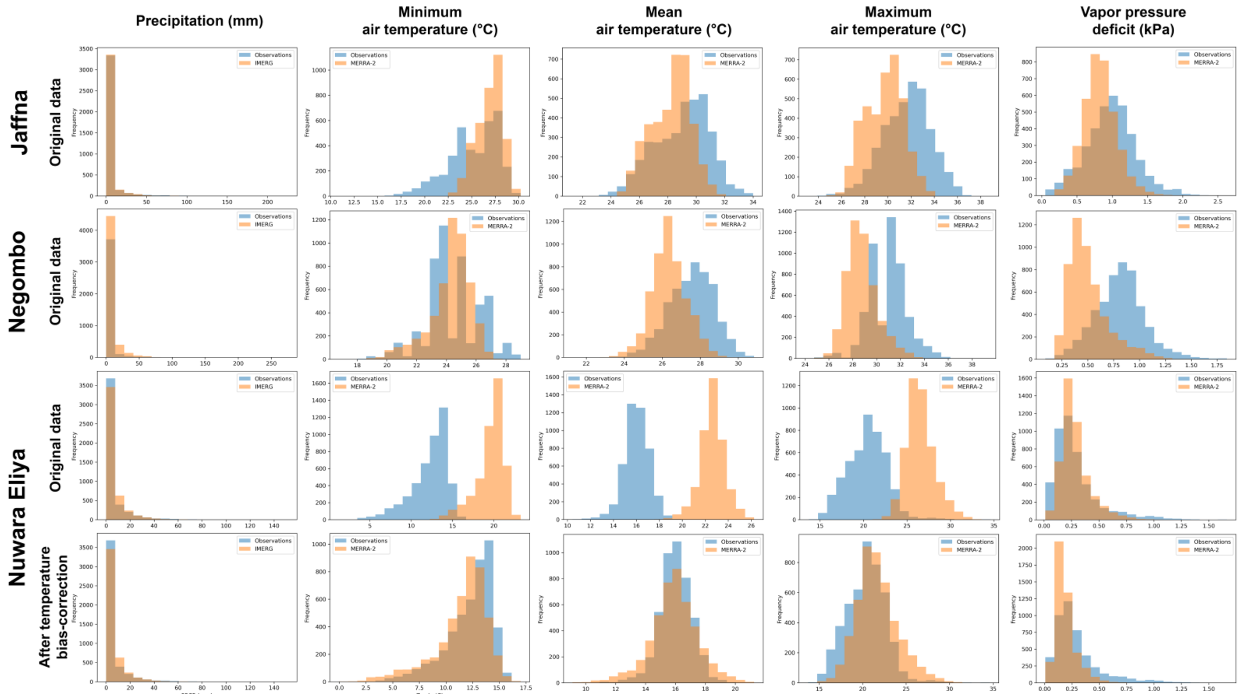

Figure S1. Comparison of MERRA-2 and IMERG meteorological data to WMO weather station observations. The first three rows (Jaffna, Negombo, Nuwara Eliya) show the original MERRA-2 and IMERG data, while the last row (Nuwara Eliya) shows the original IMERG data and the bias-corrected MERRA-2 data.

Text S2.
Step 1 – Container habitat water dynamics

Here we share further details on the first step of the modeling pipeline, simulating the water dynamics of a container habitat using the energy balance model WHATCH'EM. In particular, we show the parameter values used within WHATCH'EM to represent the container (Table S1) and the locations of interest (Table S2) and we also describe our method of estimating missing water temperature values output by WHATCH'EM (Text S2.1).

Container parameter	Variable name(s)	Value
Shape	*cshape*	"ROUND"
Radius (top)	*radius1_t*, *radius2_t*	0.131 m
Radius (bottom)	*radius1_b*, *radius2_b*	0.131 m
Height	*height*	0.368 m
Conductivity	*conduc*	0.50 W m^{-1} K^{-1}
Thickness	*thick*	0.0023 m
Reflectivity	*refl*	0.5
Shade fraction	*shade*	0.5

Table S1. Parameter values used in the WHATCH'EM model (Steinhoff et al., 2016) to represent an *Aedes* container habitat. These are the values used in Steinhoff et al. (2016) to represent a gray-colored, medium-sized bucket (18.9 L) that is half-shaded.

	Location parameter		
**Location**	**Latitude (°N)**	**Longitude (°E)**	**Elevation (m)**
Negombo	7.2008	79.8737	2.
Jaffna	9.6615	80.0255	5.
Nuwara Eliya	6.9497	80.7891	1868.

Table S2. Parameter values used in the WHATCH'EM model (Steinhoff et al., 2016) to represent this study's locations of interest: three climatically distinct cities in Sri Lanka.

Text S2.1. Estimating missing water temperature values (low water height scenario)

The WHATCH'EM container model simulates the temporal dynamics of water temperature based on energy balance equations. However, numerical stability issues arise when attempting these simulations with a low water height. Therefore, when the water height falls below a threshold of 15 mm, the model sets the water temperature and energy balance terms to a missing value and sets a constant evaporation rate of 0.02 mm/hr. If the water height then rises to a value of at least 15 mm, the model resumes normal simulation of energy balance and water temperature.

The lack of water temperature values when water height is low presents a problem for our modeling pipeline, where we are interested in using water temperature from Step 1 to calculate an uninterrupted record of development and survival rates in Step 2. In fact, it may even be *most* important to simulate water temperature values when the water height is low, since this is when the water temperature is most susceptible to large fluctuations and most likely to exceed the temperature thresholds that impact mosquito survival (i.e., when the water body has the smallest mass and therefore the lowest thermal inertia). Therefore, we have taken the water temperature outputs from WHATCH'EM and constructed an uninterrupted record of water temperature to input into Step 2 by estimating and filling in any missing water temperature values.

We tested several methods of estimating missing water temperature values and, as noted in the main text, we chose a method based on linear regressions on the MERRA-2 2-m air temperature data. All of these methods are summarized in Table S3 and described in further detail below.

Method #	Estimated quantity	Estimation method
1	Daily mean/min/max water temperature	Linear regressions against daily mean/min/max air temperature
2R	Daily min/max water temperature	Empirical equations from Focks et al. (1993) using daily min/max air temperature and sun exposure
3R	Hourly water temperature	Linear regressions against hourly air temperature
4R	Hourly water temperature	Linear regressions against hourly ground temperature

Table S3. Estimation methods tested for filling in missing WHATCH'EM water temperature values. In this work we chose to use Method 1. Rejected methods are indicated by "R".

Text S2.1.1. Estimation method 1 (used in this work)

In this method **we estimate daily mean, minimum, and maximum water temperatures based on linear regressions against daily mean, minimum, and maximum 2-m air temperature data** (from MERRA-2). Each of these three—mean, minimum, maximum—is estimated separately.

For example, consider our month-long model run for Jaffna in June 2020, where water temperature values are missing for June 18–23 and June 30 (Figure S2). To estimate missing values of mean water temperature, we first linearly regress the non-missing values of mean water temperature (***TW***) against the corresponding values of mean air temperature (***TA***), then apply this linear relationship to estimate the missing values of mean water temperature (***TW_adj***) based on the mean air temperature values for June 18–23 and June 30. We similarly estimate the missing minimum and maximum water temperature values (***TW_min_adj***, ***TW_max_adj***). We find that the estimated water temperature values look quite reasonable, being comparable to the non-missing water temperature values before and after them while also mirroring the peaks and troughs in air temperature (***TA***) and ground temperature (***TG***).

We also tested this estimation method for our month-long model run for Jaffna in April 2016 (Figure S3), which is the location and month for which WHATCH'EM produced the most missing water temperature data (61% of the timeseries). Even for this dataset, where the linear regression is based on the least amount of data and presumably performs the worst, the estimated water temperatures look reasonable. There may be overestimation of the maximum water temperature—note how the estimated maximum water temperature values (***TW_max_adj***) are about 5°C higher than the corresponding ground temperature values (***TG_max***), even though the non-missing maximum water temperature values (***TW_max***) don't differ much from the ground temperatures (***TG_max***). However, we don't expect this to be a major problem for most of our datasets, since they generally have far fewer missing values.

One remaining issue: in Step 2 of the modeling pipeline we calculate hourly development rate using *hourly* water temperature, but this estimation method only produces *daily mean*, *minimum*, and *maximum* values. To resolve this, we fill in missing *hourly* water temperature data with the estimated *daily mean* water temperature for that day.

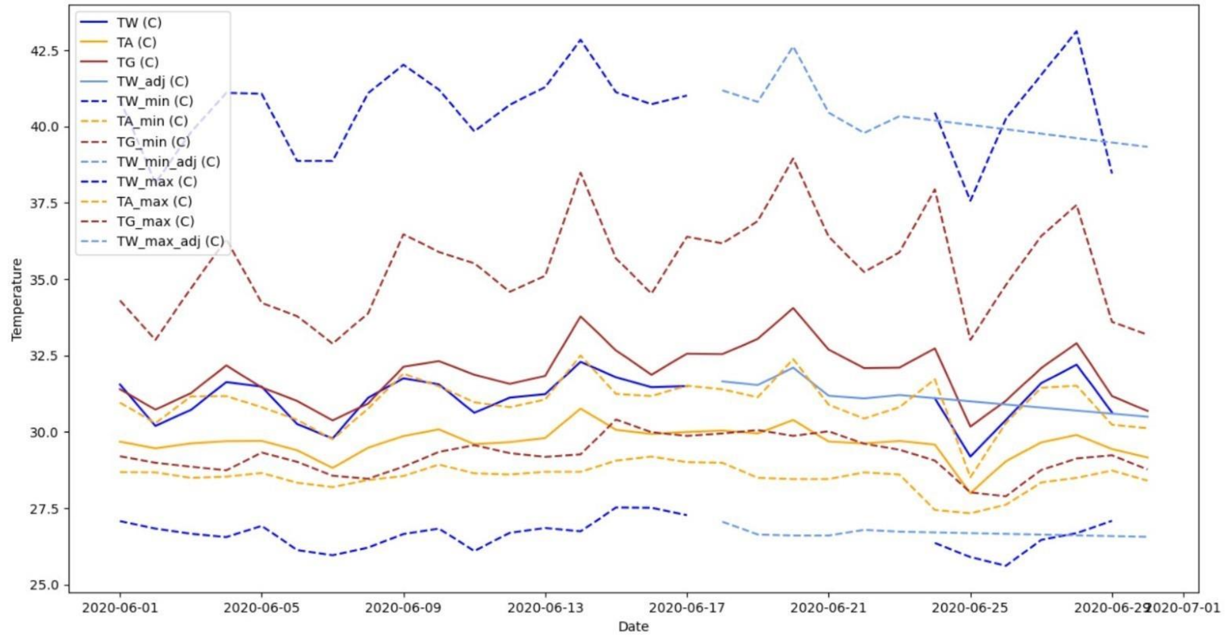

Figure S2. Daily mean, minimum, and maximum temperature values associated with a month-long WHATCH'EM model run for Jaffna in June 2020. Missing values in the water temperature timeseries produced by WHATCH'EM (***TW***) are estimated using **Estimation method 1** (***TW_adj***). The air temperature (***TA***) and ground temperature (***TG***) data used to run WHATCH'EM are from MERRA-2.

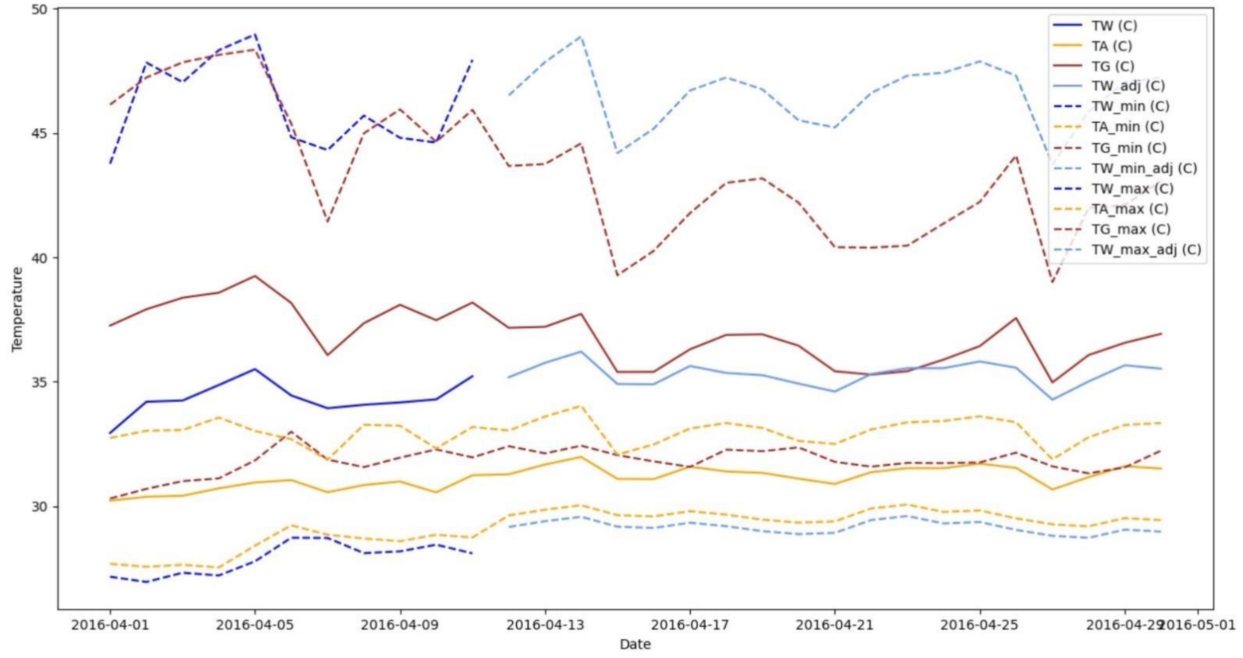

Figure S3. Daily mean, minimum, and maximum temperature values associated with a month-long WHATCH'EM model run for Jaffna in April 2016. Missing values in the water temperature timeseries produced by WHATCH'EM (***TW***) are estimated using **Estimation method 1** (***TW_adj***). The air temperature (***TA***) and ground temperature (***TG***) data used to run WHATCH'EM are from MERRA-2.

Text S2.1.2. Estimation method 2R (rejected)

In this method **we estimate daily minimum and maximum water temperatures based on empirical equations from Focks et al. (1993), which use daily minimum and maximum air temperature data and sun exposure**. The empirical equations are as follows:

$$WaterTemp_{max} = 15.03 + 0.27\ AirTemp_{min} + 0.01\ AirTemp_{max}{}^2 + 7.69\ SunExposure^2 \quad \textbf{(Equation S1)}$$

$$WaterTemp_{min} = 5.02 - 1.36\ SunExposure + 0.81\ AirTemp_{min} + 0.001\ AirTemp_{max}{}^2 \quad \textbf{(Equation S2)}$$

These empirical equations are known to be inaccurate (which in fact motivated the creation of the WHATCH'EM model), but we test them here to see if they are sufficient for estimating water temperature where WHATCH'EM cannot.

We used these equations to estimate daily minimum and maximum water temperatures using daily minimum and maximum air temperature data from MERRA-2 and a sun exposure value of 0.5 (indicating a half-shaded container, as we had specified within WHATCH'EM). We found that these estimated water temperatures differed greatly from WHATCH'EM water temperatures (compare ***TW_min*** and ***TW_max*** to ***TW_min_est*** and ***TW_max_est*** in Figure S4), so we decided that this estimation method was not effective for estimating missing water temperature values.

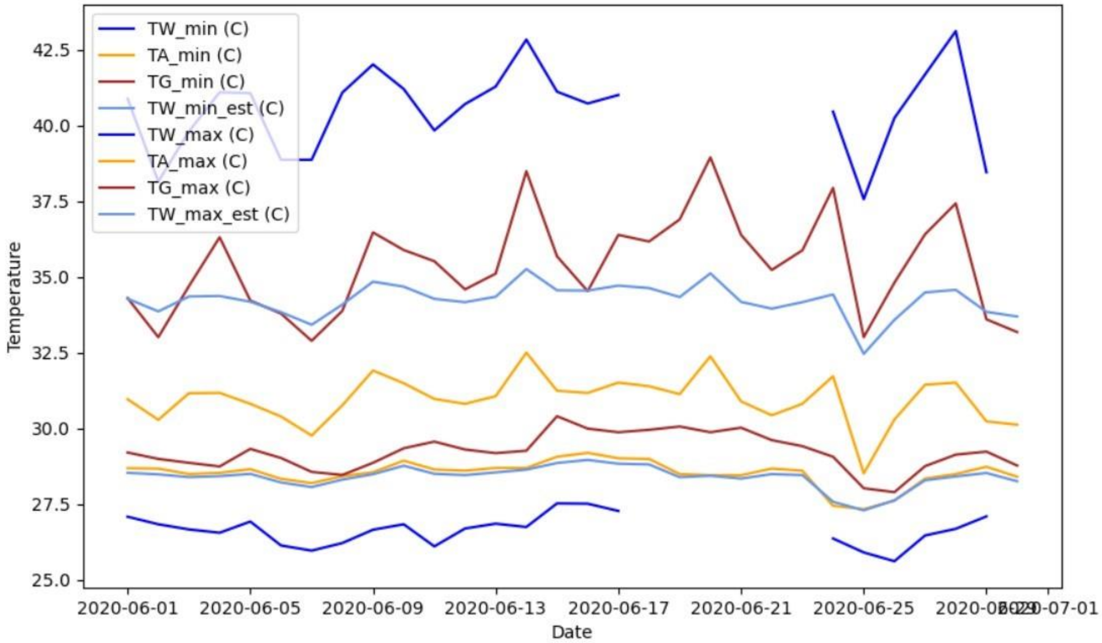

Figure S4. Daily minimum and maximum temperature values associated with a month-long WHATCH'EM model run for Jaffna in June 2020. The water temperature timeseries produced by **Estimation method 2R (*TW_min_est*, *TW_max_est*)** differs greatly from the water temperature timeseries produced by WHATCH'EM (***TW_min***, ***TW_max***). Air temperature (***TA_min***, ***TA_max***) and ground temperature (***TG_min***, ***TG_max***) are historical data from MERRA-2.

Text S2.1.3. Estimation method 3R (rejected)

In this method **we estimate hourly water temperatures based on linear regressions against hourly 2-m air temperature data** (from MERRA-2). This method is analogous to Estimation method 1, but for hourly data rather than daily mean, minimum, and maximum data. We find that the estimated water temperatures greatly underestimate diurnal fluctuations in comparison to WHATCH'EM water temperatures (compare ***TW*** to ***TW_adj*** in Figure S5), so we decided that this estimation method was not effective for estimating missing water temperature values.

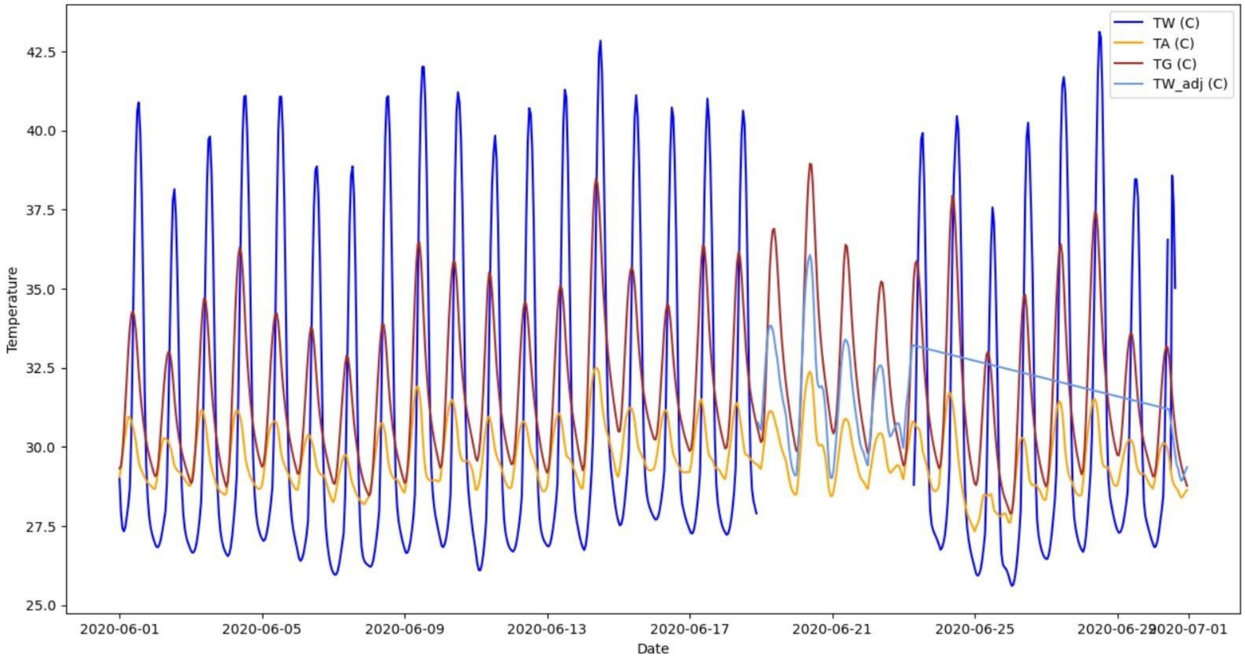

Figure S5. Daily hourly temperature values associated with a month-long WHATCH'EM model run for Jaffna in June 2020. he water temperature timeseries produced by **Estimation method 4R (*TW_adj*)** has much less diurnal variability than the water temperature timeseries produced by WHATCH'EM (***TW***). The air temperature (***TA***) and ground temperature (***TG***) data used to run WHATCH'EM are historical data from MERRA-2.

Text S2.1.4. Estimation method 4R (rejected)

In this method **we estimate hourly water temperatures based on linear regressions against hourly ground temperature data** (from MERRA-2). This method is analogous to Estimation method 1, but for hourly data rather than daily mean, minimum, and maximum data. This method is also analogous to Estimation method 3R, but with linear regression against ground temperature rather than air temperature. We find that the estimated water temperatures somewhat underestimate diurnal fluctuations in comparison to WHATCH'EM water temperatures (compare ***TW*** to ***TW_adj*** in Figure S6), so we decided that this estimation method was not effective for estimating missing water temperature values.

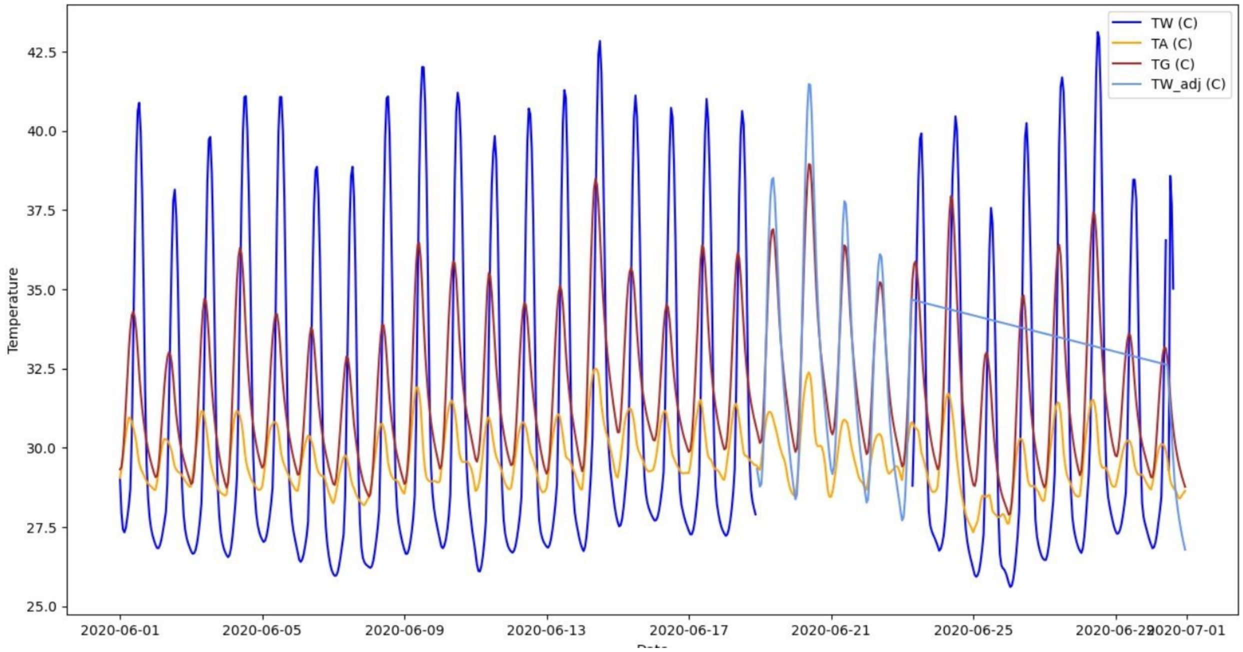

Figure S6. Daily hourly temperature values associated with a month-long WHATCH'EM model run for Jaffna in June 2020. The water temperature timeseries produced by **Estimation method 4R (*TW_adj*)** has less diurnal variability than the water temperature timeseries produced by WHATCH'EM (***TW***). The air temperature (***TA***) and ground temperature (***TG***) data used to run WHATCH'EM are historical data from MERRA-2.

Text S3.
Step 2 – Vector biology

Here we detail the second step of the modeling pipeline, which simulates development (Text S3.1) and survival (Text S3.2) of the mosquito immature stages based on models of mosquito biology. Our modeling approach borrows heavily from the mosquito population dynamics models CIMSiM (Focks et al., 1993) and Skeeter Buster (Magori et al., 2009), but notably differs in that we do not consider larval food availability and weight (i.e., we assume the larva are never food-limited) since we do not have sufficient data in our region of interest to confidently model this. For more details on the approaches of CIMSiM and Skeeter Buster, interested readers are referred to Figures 2–4 and the associated text in Focks et al. (1993) as well as Supplementary Material sections S2.2, S2.6–S2.8 in Magori et al. (2009).

Text S3.1. Development

To progress from one life stage to another, *Ae. aegypti* eggs, larvae, and pupae must first undergo sufficient growth and biological development. The hourly rate of this development is quantified by Equation S3, for which parameter values are shown in Table S4. This equation is Focks et al. (1993)'s implementation of the Sharpe & DeMichele (1977) model of development, which models *Ae. aegypti* development as a process driven by a temperature-dependent rate-controlling enzyme.

Development rate is then used to calculate cumulative development (Equation S4), which directly impacts *Ae. aegypti* growth—an *Ae. aegypti* individual must exceed a threshold of 0.95 cumulative development ($\boldsymbol{CD_t} > 0.95$) before transitioning to the next life stage (e.g., an egg hatching into a larva).

<u>Development rate</u>

$$r(T_t) = \frac{\rho_{(25°\mathrm{C})}\left(\frac{T_t}{298}\right)\exp\left[\left(\frac{\Delta H_A^{\neq}}{R}\right)\left(\left(\frac{1}{298}\right)-\left(\frac{1}{T_t}\right)\right)\right]}{1+\exp\left[\left(\frac{\Delta H_H}{R}\right)\left(\left(\frac{1}{T_{\frac{1}{2}H}}\right)-\left(\frac{1}{T_t}\right)\right)\right]} \qquad \textbf{(Equation S3)}$$

<u>Cumulative development</u>

$$CD_t = \sum_{t'=0}^{t} r(T_{t'}) \qquad \textbf{(Equation S4)}$$

$\boldsymbol{r(T_t)}$: development rate (hr^{-1}) at water temperature $\boldsymbol{T_t}$ (K)
$\boldsymbol{T_t}$: water temperature (K) at hour $\boldsymbol{t}$
$\boldsymbol{\rho_{(25°C)}}$: development rate (hr^{-1}) at 25°C assuming no temperature inactivation of the critical enzyme
$\boldsymbol{\Delta H_A^{\neq}}$: enthalpy of activation of the reaction that is catalyzed by the enzyme (cal mol^{-1})
$\boldsymbol{\Delta H_H}$: enthalpy change associated with high temperature inactivation of the enzyme (cal mol^{-1})
$\boldsymbol{T_{1/2H}}$: temperature (K) where 50% of the enzyme is inactivated from high temperature
$\boldsymbol{R}$: universal gas constant (1.987 cal mol^{-1} K^{-1})
$\boldsymbol{CD_t}$: cumulative development at the end of hour $\boldsymbol{t}$

Process	Parameter			
	$\rho_{(25°C)}$ (hr^{-1})	$\Delta H_A^{\neq}$ (cal mol^{-1})	ΔH_H (cal mol^{-1})	$T_{1/2H}$ (K)
Embryogenesis	0.01066	10,798.18	100,000.00	14,184.50
Larval development	0.00873	26,018.51	55,990.75	304.58
Pupal development	0.01610	14,931.94	-472.379.00	148.45

Table S4. Coefficients for the enzyme kinetics model of temperature-dependent development rate used by the CIMSiM mosquito population dynamics model (Focks et al. (1993); see Table 2 therein) and originally derived by Sharpe & Demichele (1977).

Text S3.2. Survival

Survival rate

Within our model, *Ae. aegypti* eggs, larvae, and pupae have a daily probability of survival (***s***). This survival rate is computed as the product of a base daily survival rate (s_0 = 0.99) and two survival factors: one associated with mortality due to water temperature extremes (s_T) and the other associated with mortality due to desiccation (s_H) (Equations S5–S6).

$$s = s_0 \cdot s_T \cdot s_H \quad \textbf{(Equation S5)}$$

$$s_0 = 0.99 \quad \textbf{(Equation S6)}$$

Temperature-based survival factor

The daily temperature-based survival factor (s_T) is the product of a survival factor based on daily maximum temperature (s_{Tmax}) and a survival factor based on daily minimum temperature (s_{Tmin}) (Equation S7). To find these two survival factors we take the maximum and minimum water temperatures for a given day (***Tmin***, ***Tmax***) and input them into a survival factor model (Equations S8–S9; Table S5; shown graphically in the main text in Figure 3). This survival factor model was developed based on laboratory experiments, as described in Focks et al. (1993).

$$s_T = s_{Tmin} \cdot s_{Tmax} \quad \textbf{(Equation S7)}$$

$$s_{Tmin} = \begin{cases} 0.05, & T_{min} \leq T_0 \\ 0.05 + 0.95\left(\dfrac{T_{min} - T_0}{T_1 - T_0}\right), & T_0 < T_{min} < T_1 \\ 1.0, & T_{min} \geq T_1 \end{cases} \quad \textbf{(Equation S8)}$$

$$s_{Tmax} = \begin{cases} 1.0, & T_{max} \leq T_2 \\ 1 - 0.95\left(\frac{T_{max} - T_2}{T_3 - T_2}\right), & T_2 < T_{max} < T_3 \\ 0.05, & T_{max} \geq T_3 \end{cases} \qquad \textbf{(Equation S9)}$$

Life stage	Temperature threshold (°C)			
	**T_0**	**T_1**	**T_2**	**T_3**
Eggs	-14	-6	30	47
Larvae/Pupae	5	10	39	44

Table S5. Thresholds for temperature-dependent survival factor. Adapted from Magori et al. (2009) (see Table S5 therein).

Desiccation-based survival factor

The daily desiccation-based survival factor (s_H) is only relevant if the container is dry: in a water-filled container this survival factor is simply 1.00. In a dry container this survival factor is a flat value for larvae (s_H = 0.05; very susceptible to desiccation) and pupae (s_H = 0.95; resilient to desiccation), while for eggs it mildly depends on vapor pressure deficit (s_H ranging from 0.95 to 0.99) (Equation S10; shown graphically in the main text in Figure 3).

$$s_H = \begin{cases} 0.99, & VPD \leq 1 \text{ kPa} \\ 0.99 - 0.04\left(\frac{VPD - 1}{3 - 1}\right), & 1 \text{ kPa} < VPD < 3 \text{ kPa} \\ 0.95, & VPD \geq 3 \text{ kPa} \end{cases} \qquad \textbf{(Equation S10)}$$

VPD : vapor pressure deficit (kPa)

Text S4.
Step 3 – Vector population dynamics

Here we expand on the third step of the modeling pipeline, which simulates population dynamics of *Ae. aegypti* eggs, larvae, and pupae. We detail the algorithms used to do so (Text S4.1; Figures S7–S9) and describe our method of minimizing variance in the outputs of the modeling pipeline that arise solely from the stochasticity inherent in the model (Text S4.2).

Text S4.1. Vector population dynamics algorithms

The algorithms we used to model population dynamics, which depend on both the development and survival rates from Step 2 and on other environmental and probabilistic factors, determine an *Ae. aegypti* individual's development, survival, and possible transition to the next life stage on each simulation day. The flowcharts presented here show the steps of these algorithms (Figures S7–S9). These algorithms borrow heavily from the mosquito population dynamics models CIMSiM (Focks et al., 1993) and Skeeter Buster (Magori et al., 2009). For details on their approaches, interested readers are referred to Figure 1 and the "Biological Relationships" section of Focks et al. (1993) and the "Local population dynamics of immature and adult *Ae. aegypti*" section of Magori et al. (2009).

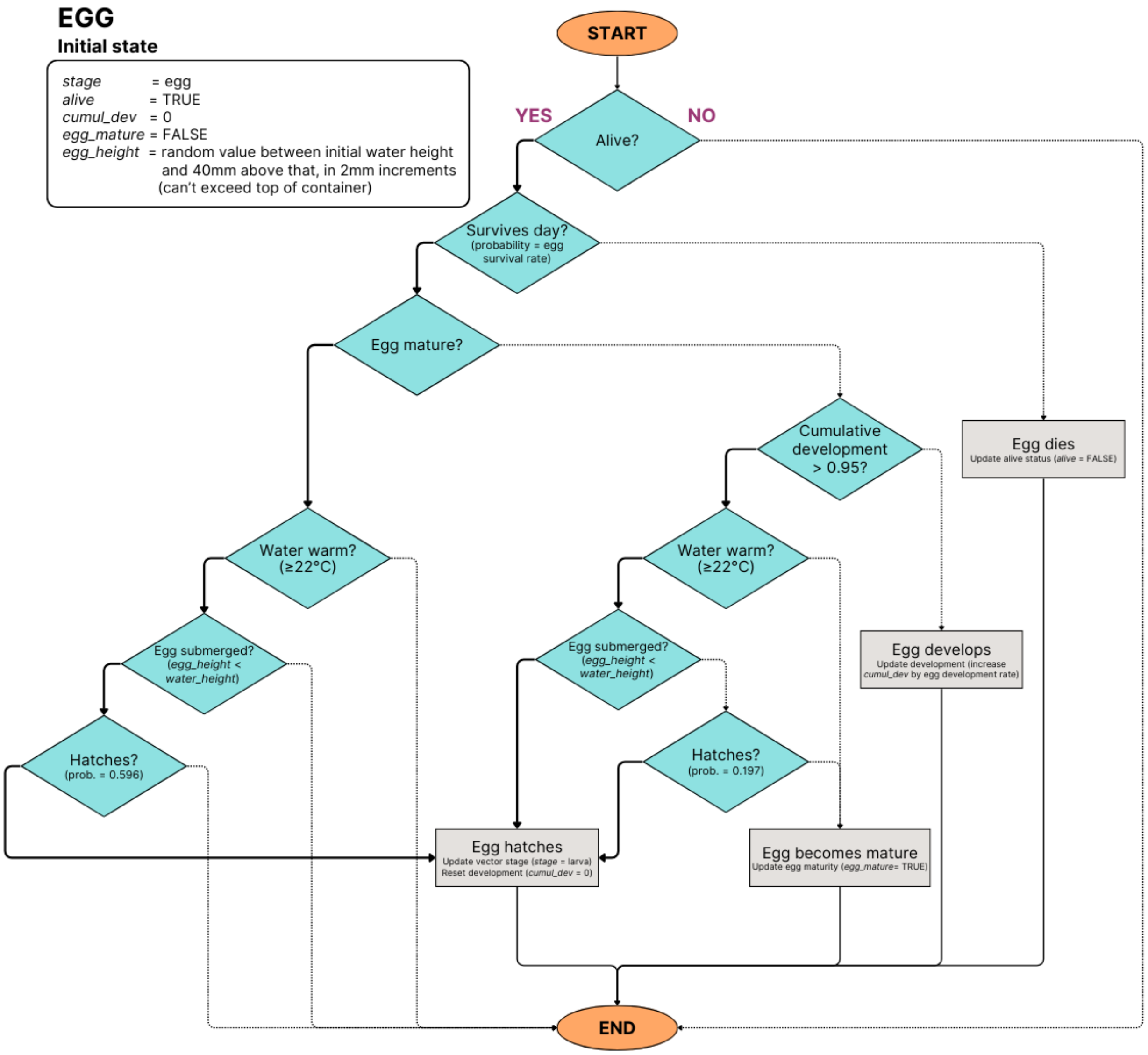

Figure S7. Algorithm for egg development/hatching. At each condition check (diamond), the thicker, solid arrow on the left represents a YES and the thinner, dotted arrow on the right represents a NO. This figure is adapted from Magori et al. (2009) (see Figure 2 therein).

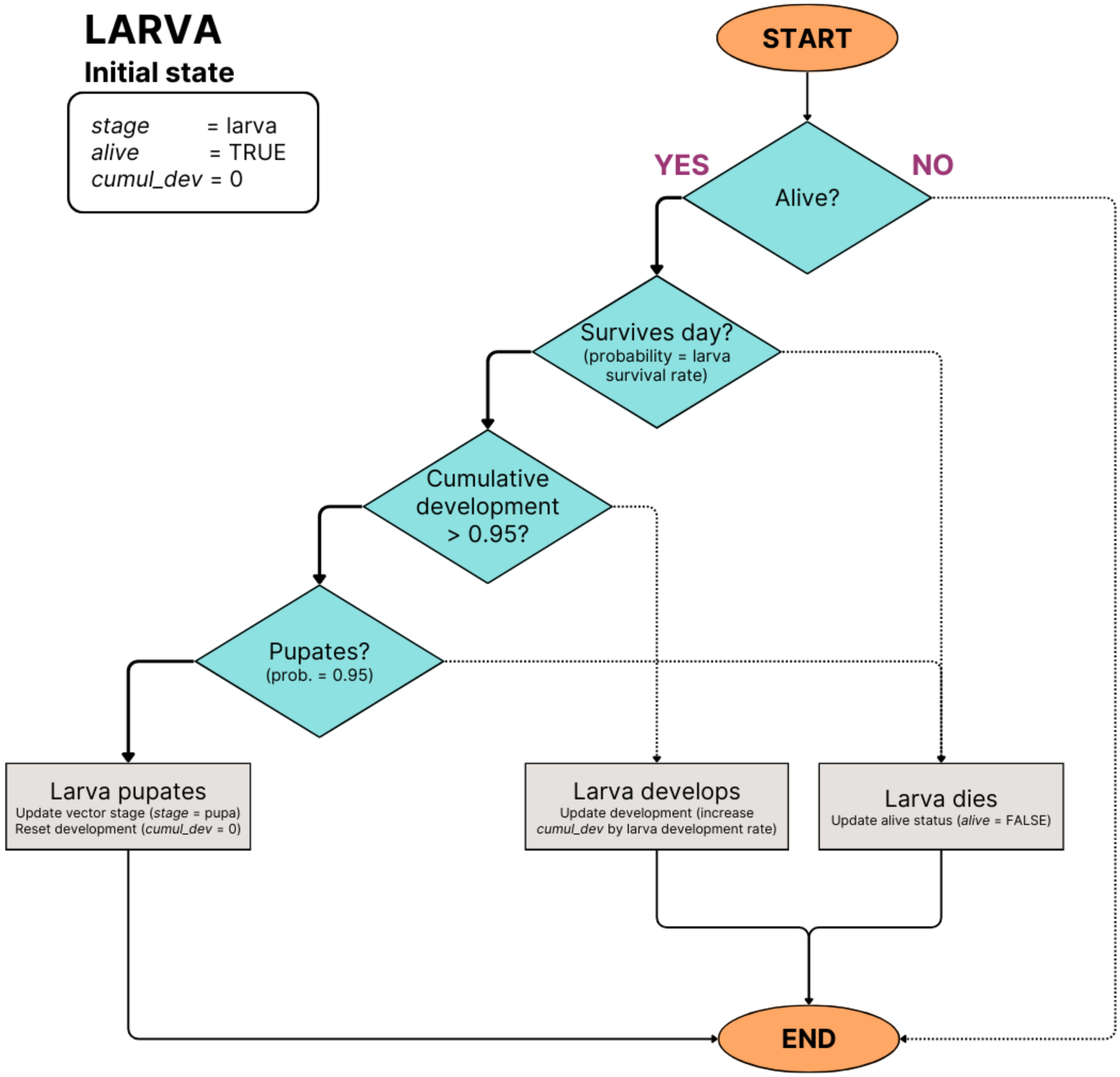

Figure S8. Algorithm for larva development/pupation. At each condition check (diamond), the thicker, solid arrow on the left represents a YES and the thinner, dotted arrow on the right represents a NO.

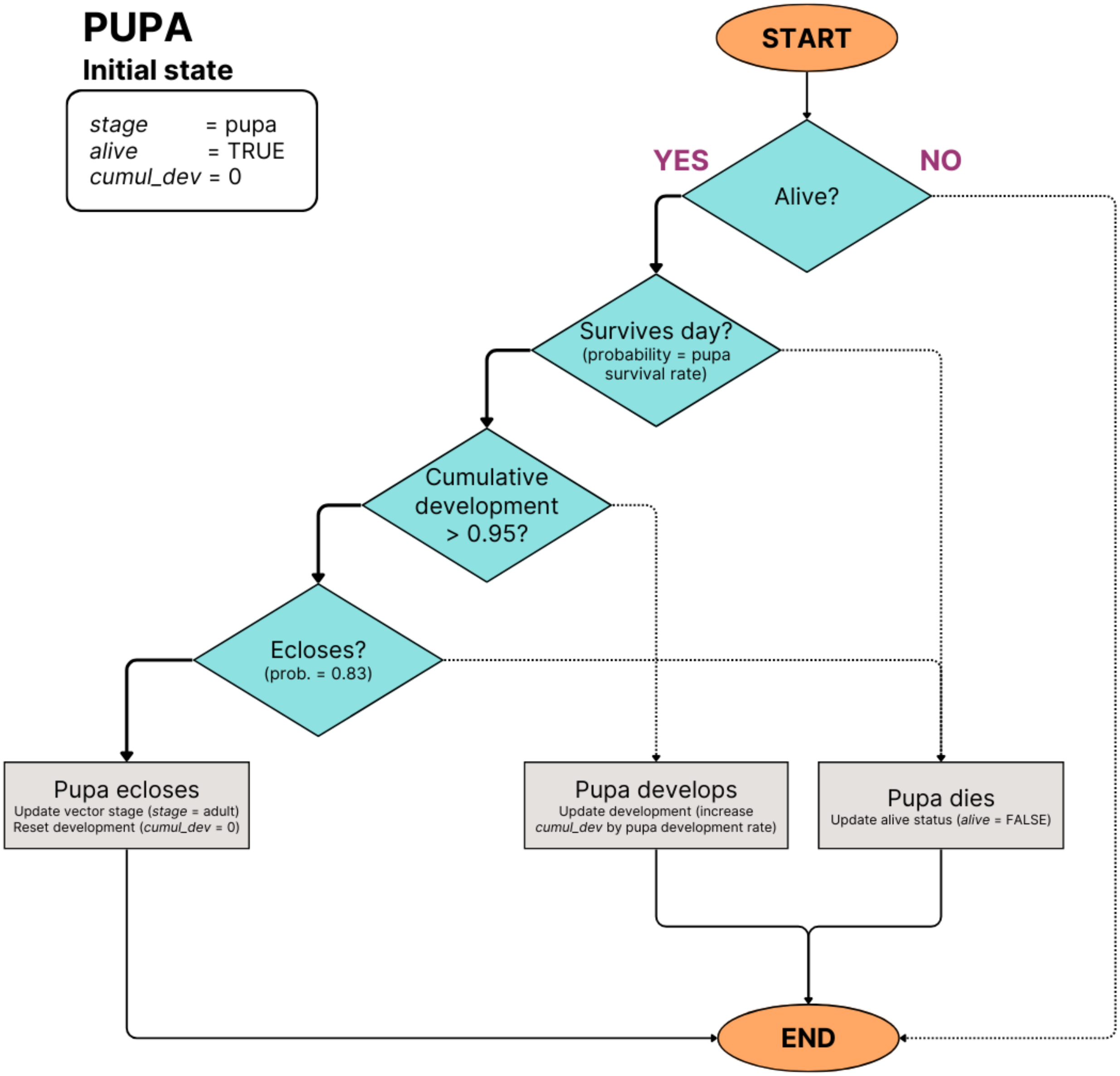

Figure S9. Algorithm for pupa development/pupation. At each condition check (diamond), the thicker, solid arrow on the left represents a YES and the thinner, dotted arrow on the right represents a NO.

Text S4.2. Minimizing variability in simulated adult population due to stochasticity

Our population dynamics model is stochastic since each *Ae. aegypti* individual's hatching, pupation, and eclosion succeeds or fails probabilistically (Figures S10–S15). This means model runs with identical climate inputs and initial conditions will typically yield different outputs of simulated adult population.

This presents an issue given the aim of our work: we want to be able to wholly attribute the variance in simulated adult population to climatic interannual variability. Therefore, we aim to run our model such that we minimize any variance in the results that is due to the model's randomness. We do so by conducting each model run with a *large enough* initial populations of eggs, larvae, and pupae to average over the stochasticity: for example, whether a single egg happens to hatch or die should *negligibly* influence the average life history of the population as a whole.

The question then becomes, what is a *large enough* initial population so that this is true? As noted in the main text, we decided that **1000 individuals** of each life stage (eggs, larvae, pupae) was sufficient. We chose this population size based on testing of our model as described below.

We tested the population dynamics model with two different initial populations sizes: 100 and 1000 individuals. We ran the model for each location (Jaffna, Negombo, Nuwara Eliya) and year (2001–2020), for the specific calendar month in which the simulated adult population for that location had the lowest interannual variability (based on previous testing): Jaffna – December; Negombo – July; Nuwara Eliya – June. For each of these parameter combinations (initial population, location+month, year) we conducted 10 model runs and made box plots to show the variance among runs (Figures S10–S15).

Our aim here is to assess whether the variance in simulated adult population across years is a clear signal that is not obfuscated by the variance due to model randomness. First, let's look at our results for an initial population size of 100 individuals (Figures S10, S12, S14). We see that the differences in simulated adult population across years (our signal of interest) often falls within the range of variability due to model randomness. For example, for Jaffna (Figure S10) the year 2004 seems more favorable for higher adult populations than 2003, yet there is an overlap in their boxplots: due to the randomness in the models, there's a decent chance that a given model run for 2004 and for 2003 would yield *lower* simulated adult population in 2004.
This suggests that an initial population size of **100 individuals *is not* sufficient** for minimizing the variance due to the model's stochasticity.

Looking at our results for an initial population size of 1000 individuals Figures S11, S13, S15), we see better separation of the simulated adult population across years. Looking again at Jaffna in December (Figure S11), the year 2004 is definitively more favorable for adult populations than 2003, with a wide gap between their boxplots. We note that for Nuwara Eliya (Figure S15) it might appear that interannual variability is masked by the variability due to randomness, but consider that the simulated population values are mostly varying in a very small range (~50 individuals out of ~1400). Therefore this isn't a concern; there's simply not much interannual variability here (aside from the data for 2011).
This testing suggests that an initial population size of **1000 individuals *is* sufficient** to minimize the variance due to the model's stochasticity—the variability due to changing climate conditions from year to year is not overshadowed by the variability due to the model's randomness.

Jaffna (December)

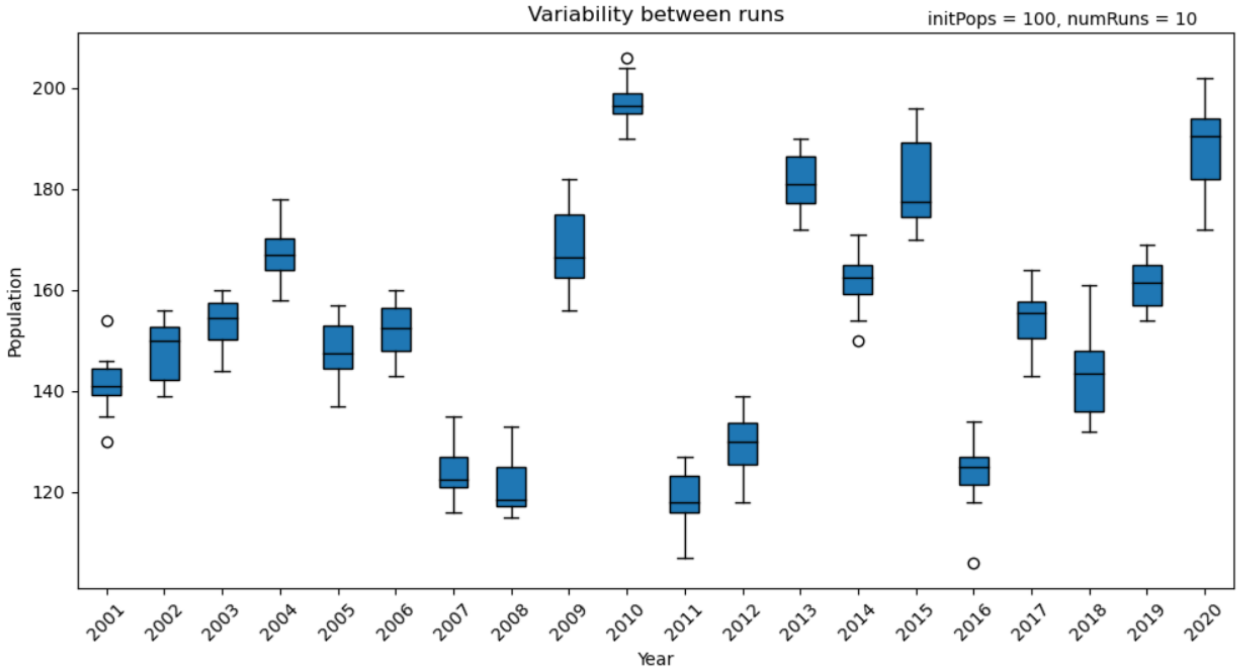

Figure S10. Variability in simulated adult population for **Jaffna in December**, given initial populations of **100** eggs, larvae, and pupae. Each box plot represents 10 model runs with identical initial conditions.

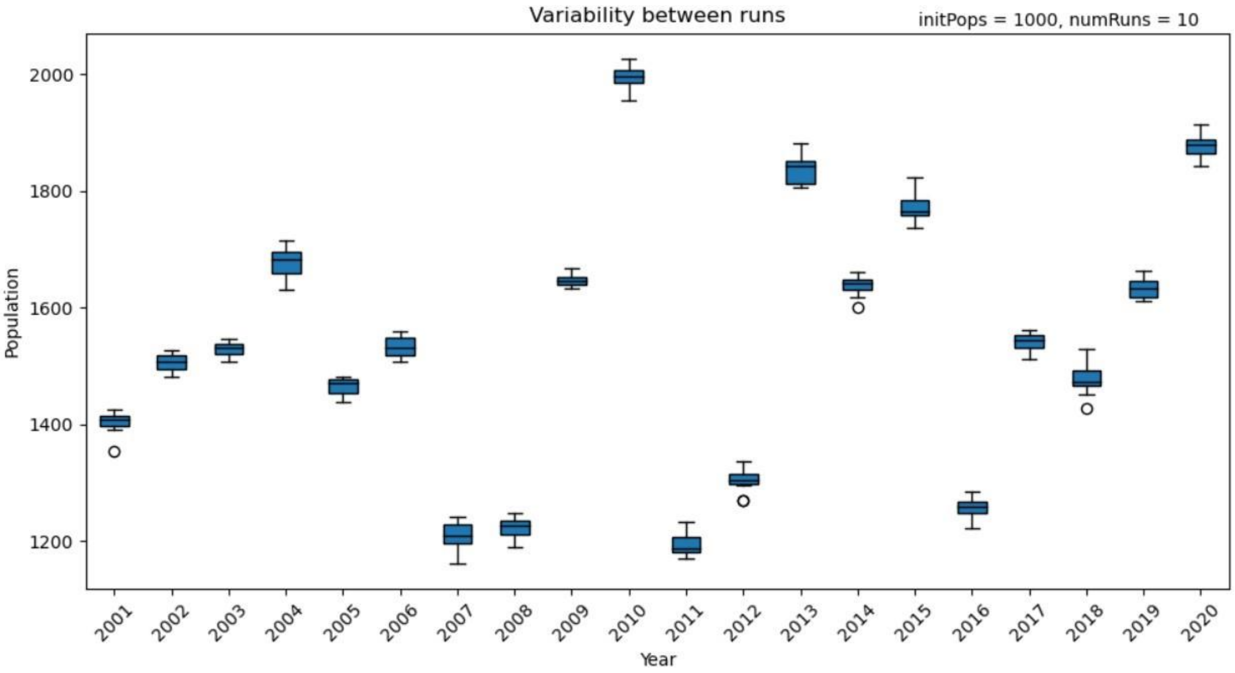

Figure S11. Variability in simulated adult population for **Jaffna in December**, given initial populations of **1000** eggs, larvae, and pupae. Each box plot represents 10 model runs with identical initial conditions.

Negombo (July)

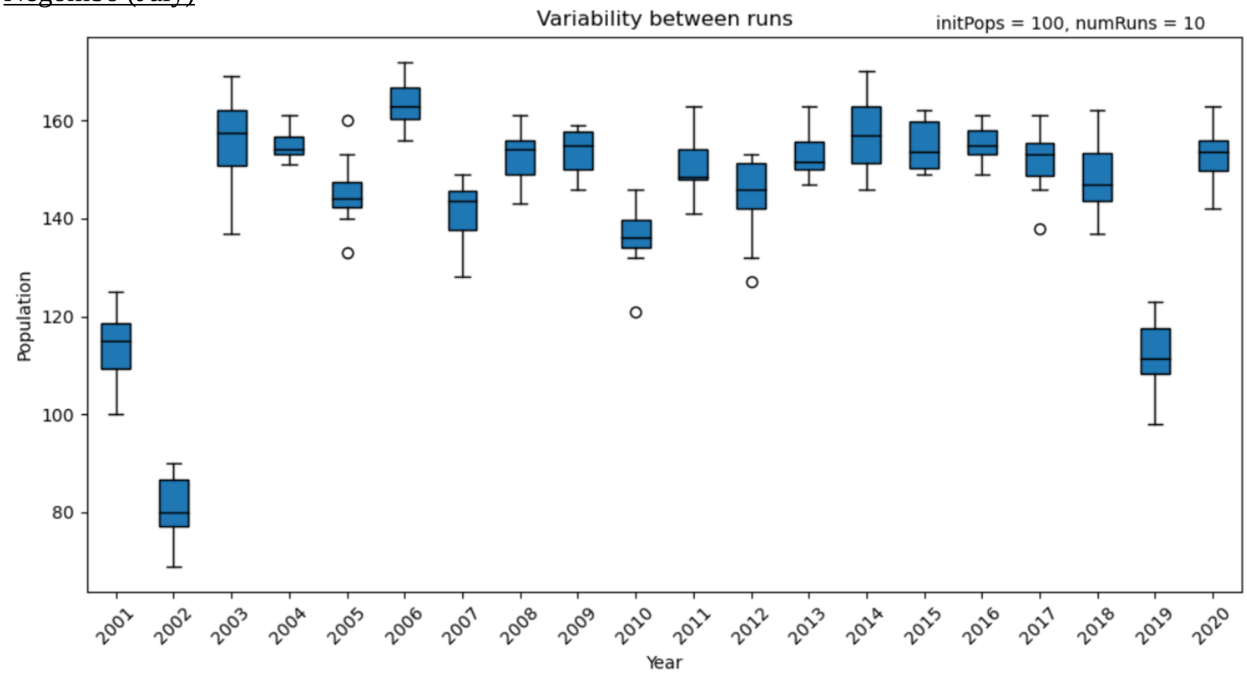

Figure S12. Variability in simulated adult population for **Negombo in July**, given initial populations of **100** eggs, larvae, and pupae. Each box plot represents 10 model runs with identical initial conditions.

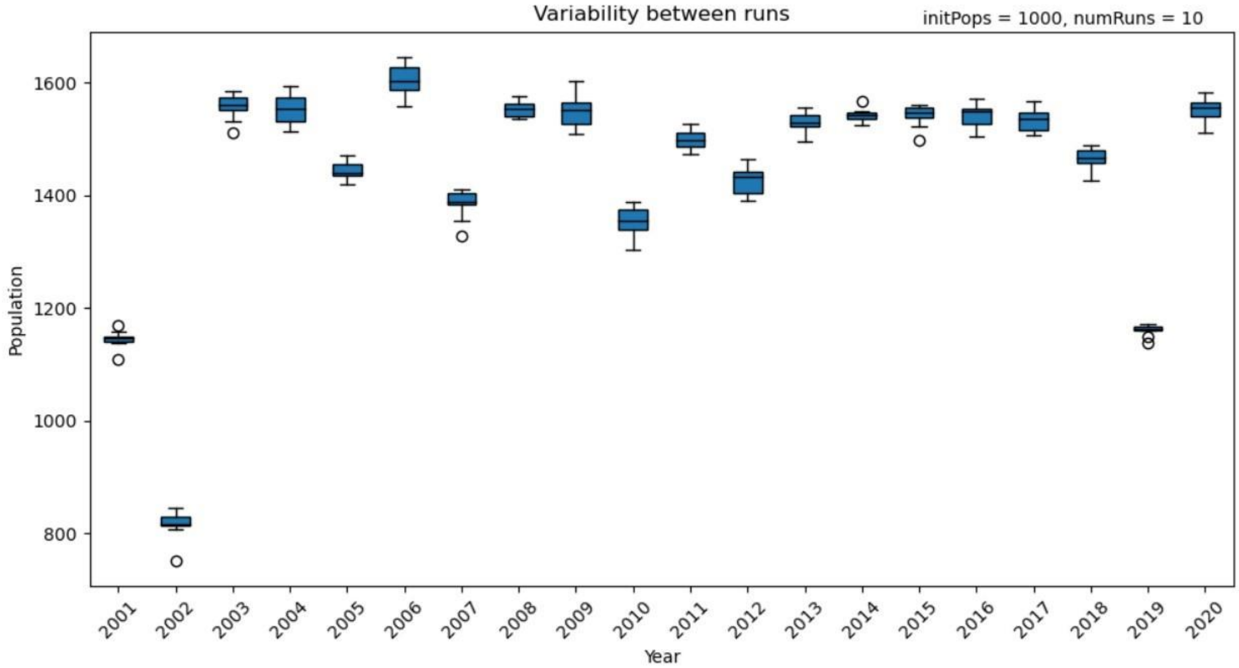

Figure S13. Variability in simulated adult population for **Negombo in July**, given initial populations of **1000** eggs, larvae, and pupae. Each box plot represents 10 model runs with identical initial conditions.

Nuwara Eliya (June)

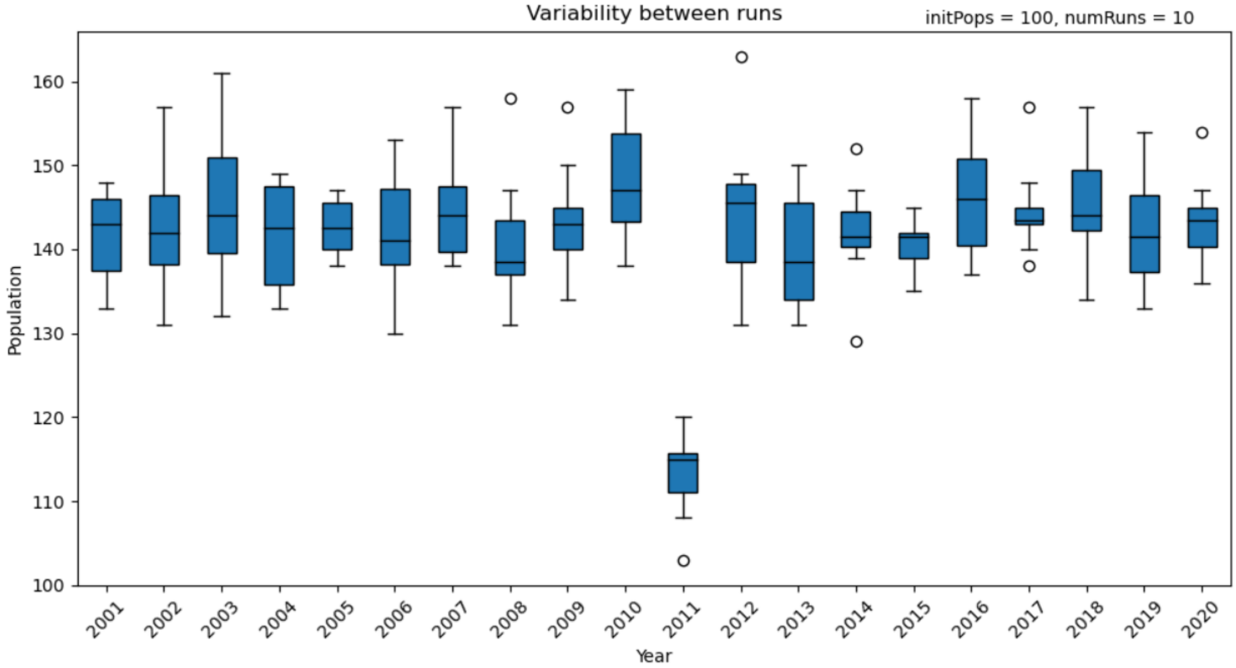

Figure S14. Variability in simulated adult population for **Nuwara Eliya in June**, given initial populations of **100** eggs, larvae, and pupae. Each box plot represents 10 model runs with identical initial conditions.

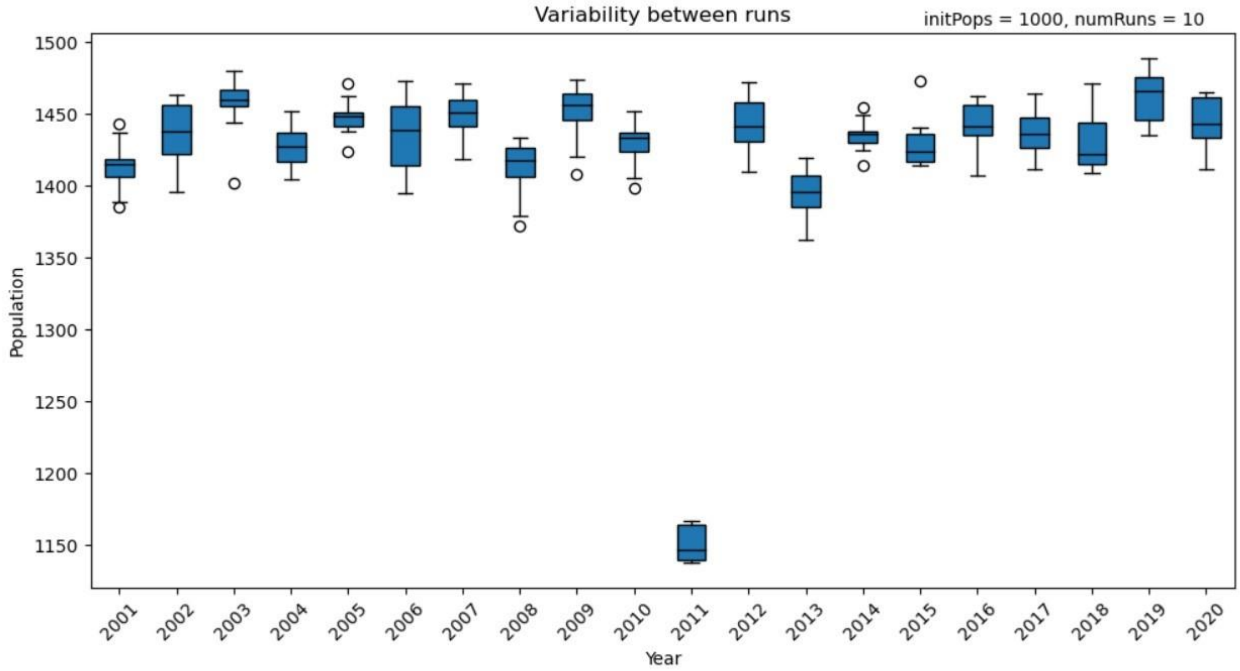

Figure S15. Variability in simulated adult population for **Nuwara Eliya in June**, given initial populations of **1000** eggs, larvae, and pupae. Each box plot represents 10 model runs with identical initial conditions.

Text S5.
Additional supporting figures

Here we present additional supporting figures referenced by the main text.

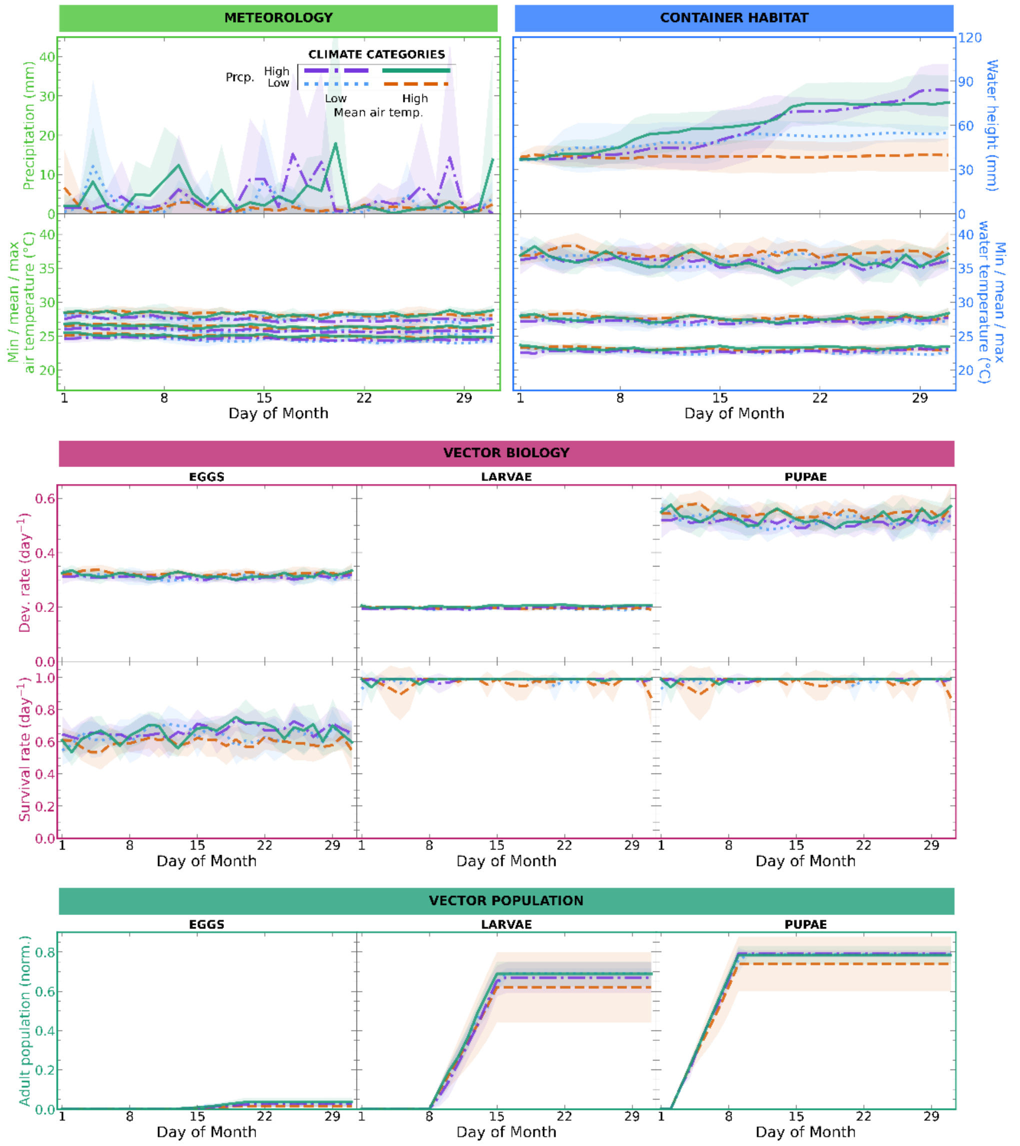

Figure S16. Timeseries of modeling pipeline input/output variables from all **July model runs for Negombo** (2001–2020) for different climate categories. All variables are daily values. The variables are as follows: (meteorology) precipitation, minimum/mean/maximum 2-m air temperature; (container habitat dynamics) water height, minimum/mean/maximum water temperature; (vector biology) development and survival rate for eggs, larvae, pupae; (vector population dynamics) population of adults from eggs, larvae, pupae. Each of the four climate categories represents a subset of the twenty years of data, sorted according to combinations of lower/upper quantiles of monthly mean 2-m air temperature and lower/upper quantiles of monthly total precipitation. In each plot, each timeseries and its shading represents the mean and standard deviation across the years of data within a climate category: low air temperature + low precipitation (dotted blue line), high air temperature + low precipitation (dashed orange line), low air temperature + high precipitation (dash-dotted purple line), high air temperature + high precipitation (solid green line).

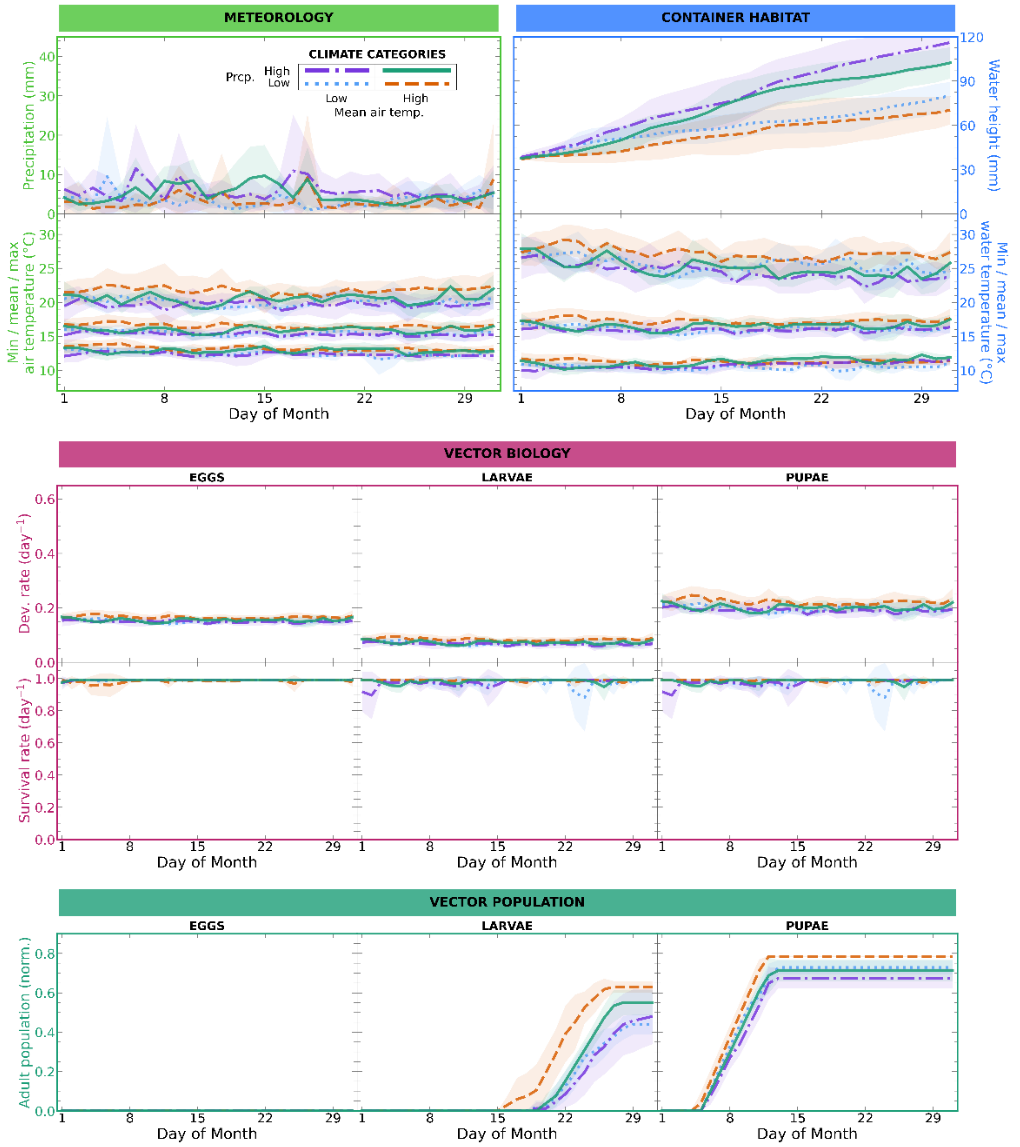

Figure S17. The same as **Figure S16**, but for all **July model runs for Nuwara Eliya** (2001–2020) for different climate categories.

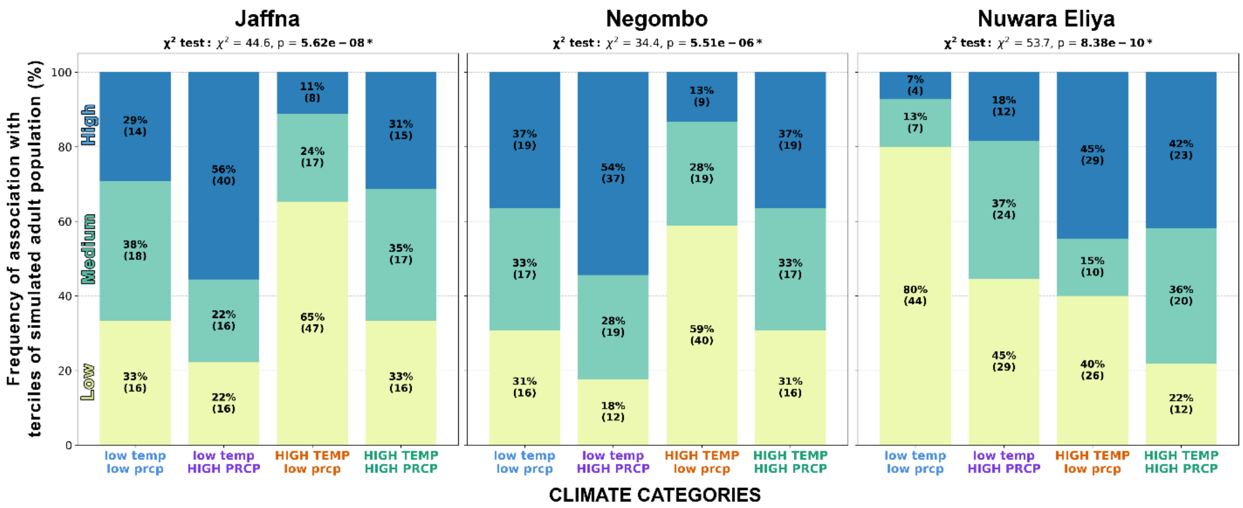

Figure S18. The associations between categories of monthly climate conditions and **simulated monthly adult population from larvae**. The four categories of climate conditions are combinations of lower/upper quantiles of monthly mean 2-m air temperature and lower/upper quantiles of monthly total precipitation. The three categories of adult population from pupae (shown as the differently colored bars) are low, medium, and high terciles of population at end of month. Annotations within each bar indicate the amount of monthly adult population data points both as a percentage of all data points within that climate category and as a count of data points. The chi-square test result shows a bolded p-value if p-value < 0.05 (i.e., when we consider that the two categorical variables, climate categories and simulated adult population terciles, are not independent). These plots show data aggregated from all months of the year, but the categorization was done separately for each calendar month to minimize the impact of seasonality.

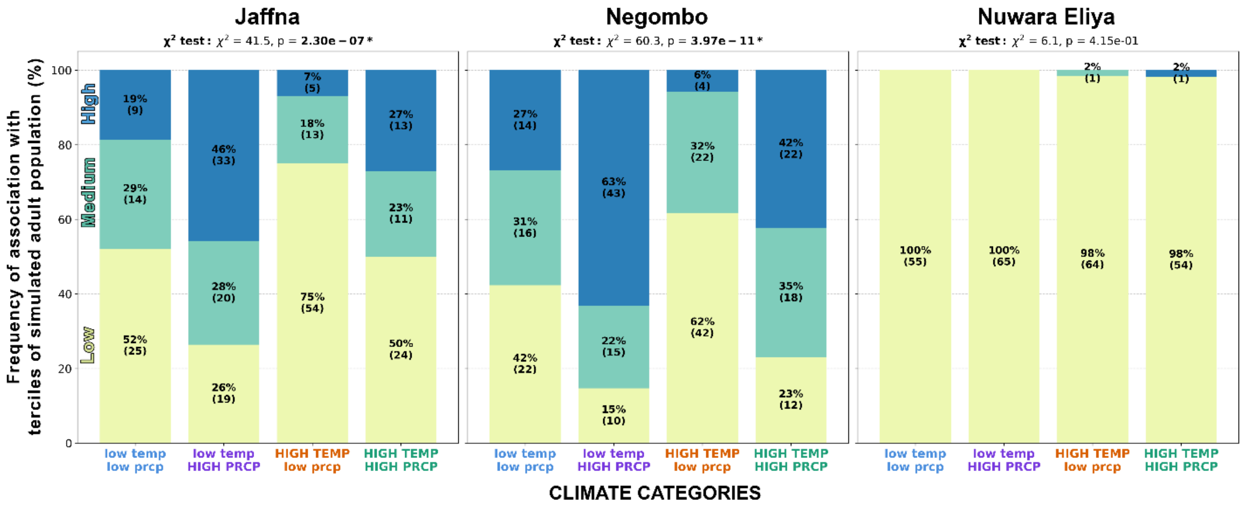

Figure S19. The same as **Figure S18**, but for associations between categories of monthly climate conditions and **simulated monthly adult population from eggs**. For Nuwara Eliya almost all model runs yielded zero simulated adult populations, which we categorized into the "low" tercile.

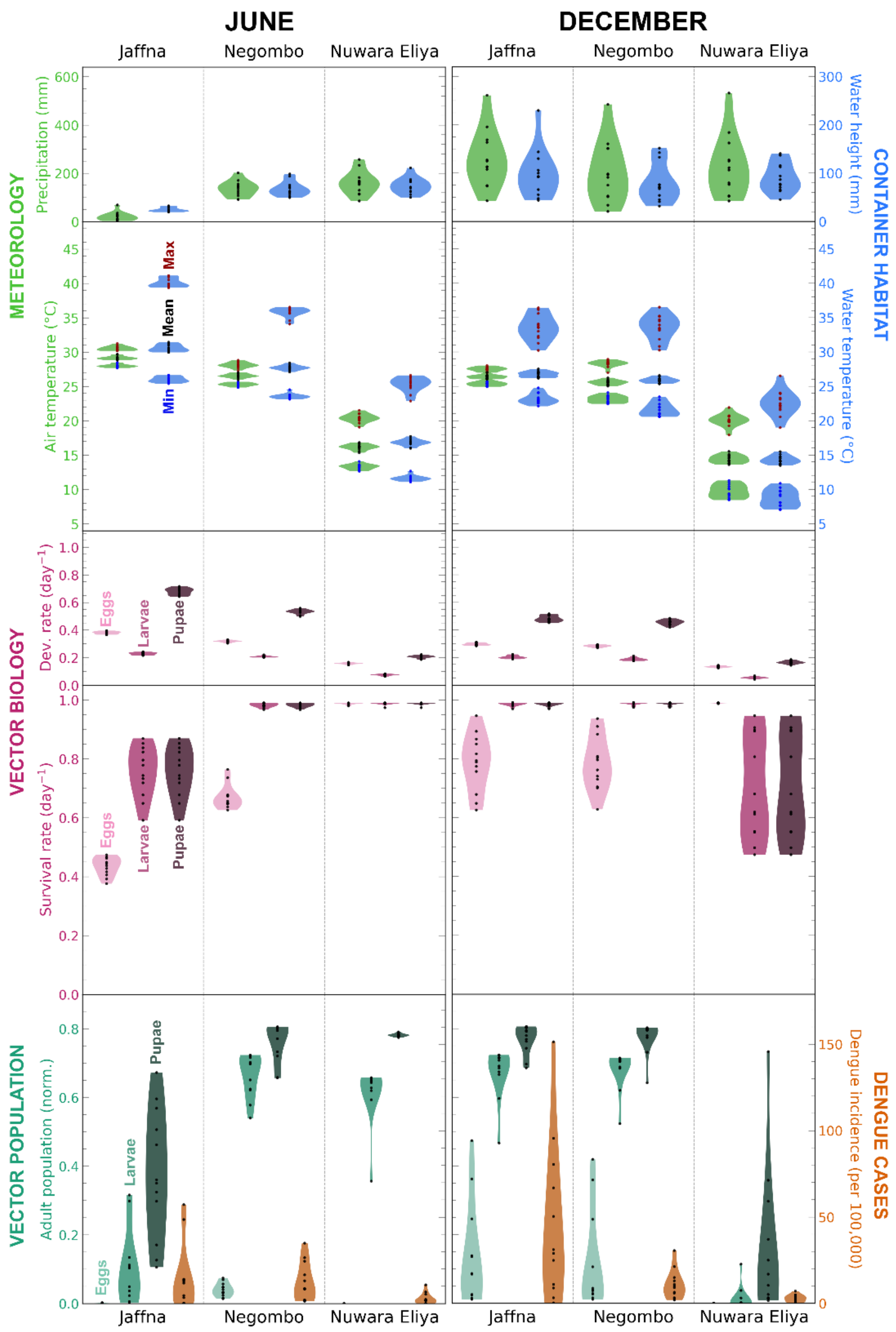

Figure S20. Violin plots of modeling pipeline input/output variables and recorded dengue incidence for Jaffna, Negombo, and Nuwara Eliya for the months June and December (2007–2020, excluding outbreak years 2017 and 2019). All variables have been resampled to monthly values such that each violin plot contains one datapoint for each year of data. The variables are as follows: (meteorology) total precipitation, minimum/mean/maximum 2-m air temperature; (container habitat dynamics) mean water height, minimum/mean/maximum water temperature; (vector biology) mean development and survival rate for eggs, larvae, pupae; (vector population dynamics) population of adults from eggs, larvae, pupae at end of month, normalized to initial population.

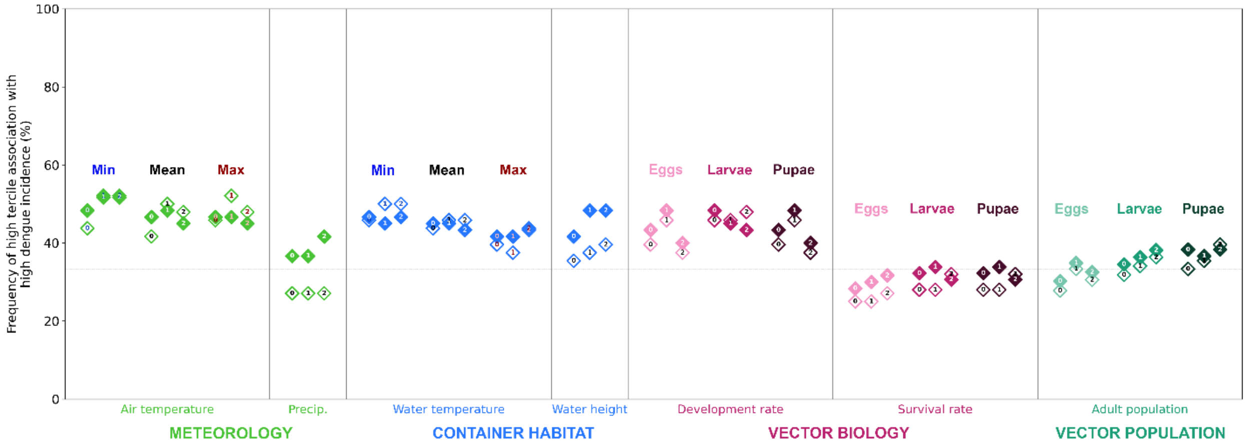

Figure S21. The frequency of association between the high tercile of dengue incidence and the high tercile of each modeling pipeline input/output variable for **Jaffna** (2007–2020). The modeling pipeline variables (all resampled to monthly values) are as follows: (meteorology) minimum/mean/maximum 2-m air temperature, total precipitation; (container habitat dynamics) minimum/mean/maximum water temperature, mean water height; (vector biology) mean development and survival for eggs, larvae, pupae; (vector population dynamics) population of adults from eggs, larvae, pupae at end of month. For each modeling pipeline variable there are six data points corresponding to combinations of different lag times (0, 1, and 2 months; indicated by the number within the data point) and different years of data (including or excluding the outbreak years of 2017 and 2019; indicated by a filled or not filled data point, respectively). The dashed line indicates the expected frequency of association if the associations among terciles were random (33%).

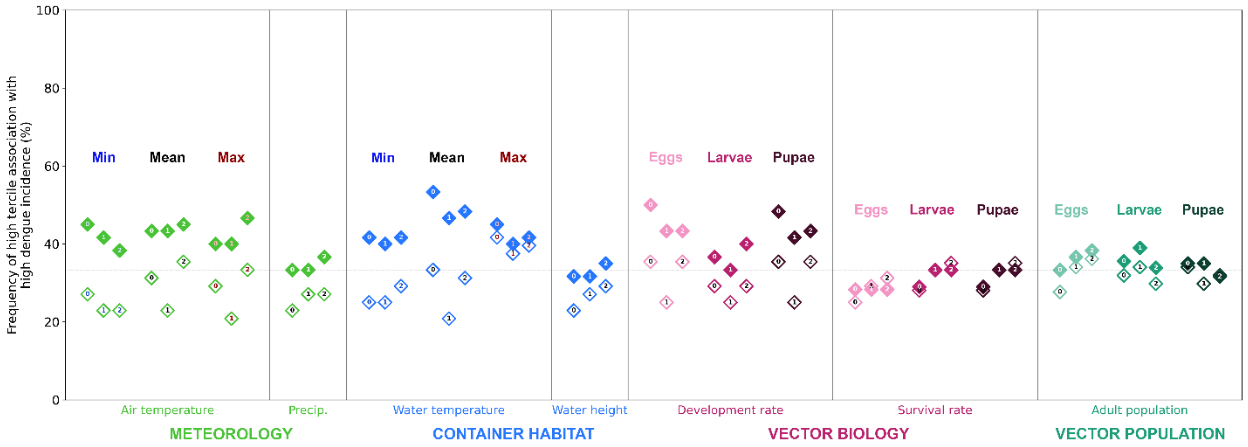

Figure S22. The same as **Figure S21**, but for **Negombo**.

Text S6.
Testing modeling pipeline sensitivity to choice of temperature-dependent survival model

We ran the modeling pipeline with two different temperature-dependent survival models, testing how sensitive our results are to this choice.

The primary temperature-dependent survival model (i.e., what we focus on in the main text) is described in detail in Section 2.2.3 of the main text and Text S3.2 here in the Supporting Information, and is shown graphically in Figure 3 (the left inset plot). We will hereafter refer to that temperature-dependent survival model as the *primary model*.

In this section we describe the second temperature-dependent survival model that we tested—hereafter referred to as the *alternate model*. We explain how we constructed this alternate model and show the resulting outputs from the modeling pipeline.

Text S6.1. Constructing the alternate model

We constructed an alternate model of temperature-dependent survival based on compiled data from Eisen et al. (2014). This paper is one of several cited by Mordecai et al. (2017) in their compilation of mosquito thermal biology data, and we chose to use the Eisen et al. (2014) data because it, unlike other works, provides survival data differentiated by life stage (i.e., eggs, larvae, pupae). These seemed the most appropriate data to use for comparison to the primary model, which also has separate survival curves for each immature life stage.

The Eisen et al. (2014) data is in the form of scatter plots of survival probability over a whole life stage versus temperature (Figures 1B, 2B, and 3B in Eisen et al., 2014), and we used several data processing steps to extract survival curves compatible with our modeling pipeline.

1. Convert Eisen et al. (2014) datapoints from *whole-stage* survival probability to *daily* survival probability.
   For each datapoint's temperature, we found the corresponding development rate (see Text S3.1) and estimated life stage duration as the days, *d*, it would take to exceed a cumulative development of 0.95. We then estimated daily survival probability as the dth root of the whole-stage survival probability. For example, if the development rate at said temperature is 0.475, then the estimated life stage duration is 2 days (0.95/0.475) and the daily survival probability is given by $(\textit{whole-stage survival probability})^{1/2}$.

2. Fit the daily survival probability data using a quadratic function.
   This is similar to the approach of Mordecai et al. (2017) for fitting temperature-sensitive traits. For the fit we excluded survival probability data at extreme low or high temperatures where survival was zero (e.g., temperatures <10°C or >38°C in Figure S25) We fit the remaining survival probability data (e.g., 10°C ≤ temperature ≤ 38°C in Figure S25) using a quadratic function of the form $-c(T–T0)(T–Tm)$, where c is a positive rate constant, T is daily mean temperature, T_0 is the minimum temperature at which survival probability is nonzero, and T_m is the maximum temperature at which survival probability is nonzero (i.e., T_0 and T_m are the zeros of the quadratic fit).

3. Clip the quadratic fit so that survival probability values are bounded between 0 and 1.

The resulting survival curves are shown in Figures S23–S25 alongside the primary model's survival curves for comparison. Note that the alternate model's survival curves are quite different from the primary model's survival curves, with survival values being above the minimum value across a much narrower range of temperatures (e.g., in Figure S23: 7°C to 36°C for the alternate model vs. -14°C to 47°C for the primary model). This *does not necessarily* indicate that the two models will produce different survival rates because the two sets of survival curves are meant to be interpreted in two different ways: the primary model is used with daily minimum and maximum temperature (as described in Text S3.2), while the alternate model is used with daily mean temperature.

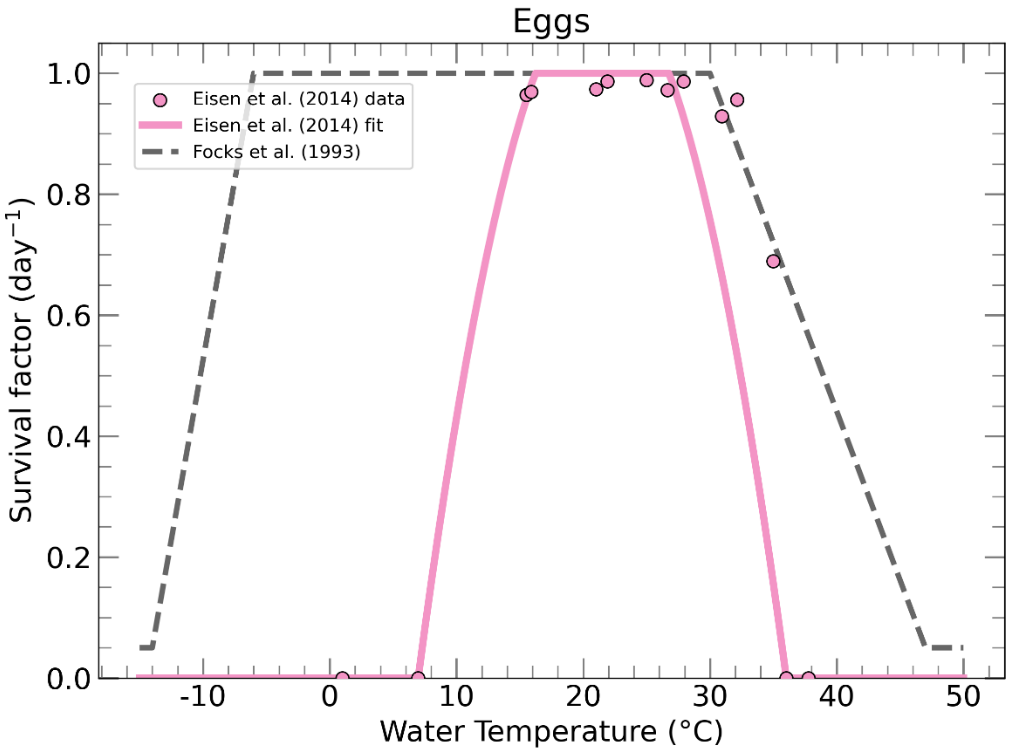

Figure S23. Temperature-based survival factors for **eggs**, including the original data compiled by Eisen et al. (2014) (colored circles), our clipped quadratic fit to that data (solid colored line), and the survival curve from the primary model (dashed gray line).

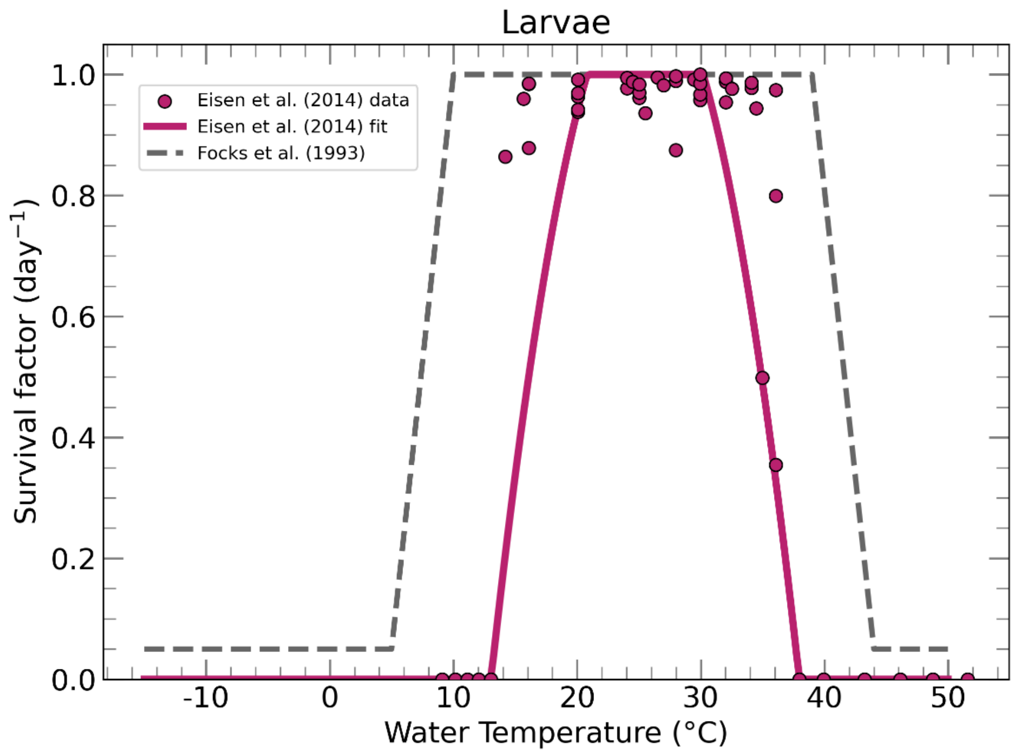

Figure S24. The same as **Figure S23**, but for **larvae**.

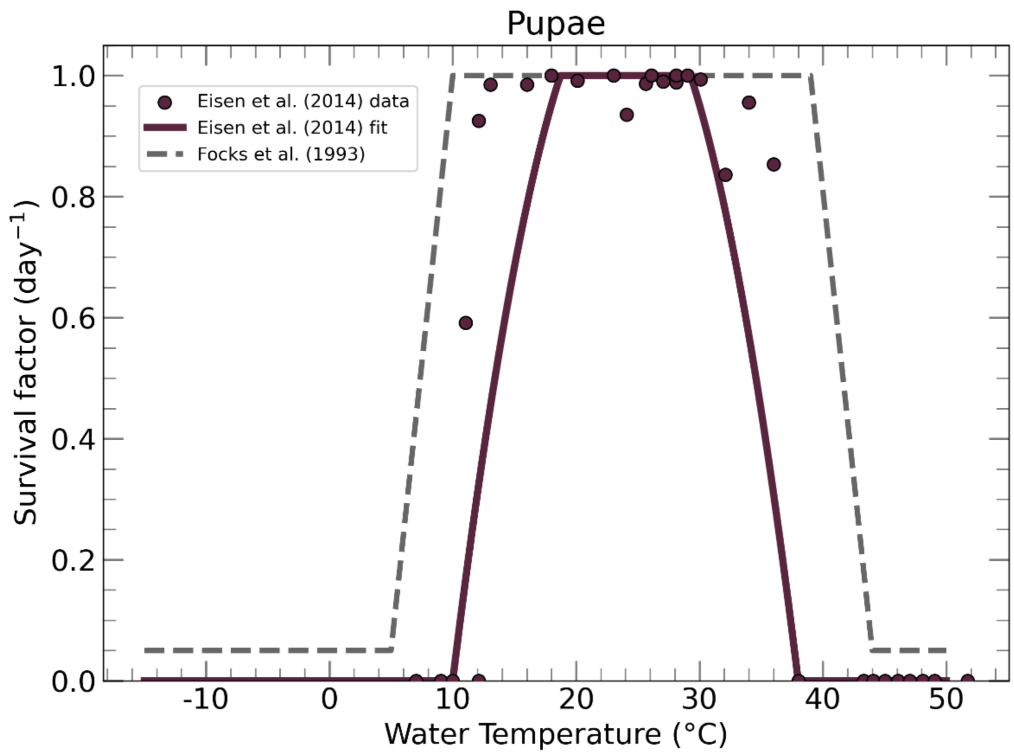

Figure S25. The same as **Figure S23**, but for **pupae**.

Text S6.2. Assessing modeling pipeline outputs yielded by the alternate model

Here we compare the modeling pipeline outputs when using the alternate model's survival curves to the outputs when using the primary model's survival curve (Figures S26–S31). Key similarities and differences in the outputs due to the alternate model are as follows:

- The survival rates in Jaffna and Negombo are generally higher, while the survival rates in Nuwara Eliya are lower (Figures S26–S31).

- The high tercile-high tercile associations between simulated adult population and dengue incidence are generally comparable (Figures S29–S31).

- Seasonal dynamics of dengue incidence are generally captured by the simulated adult population, but…
  - …for Jaffna and Negombo the simulated adult populations don't capture the dip in dengue incidence around September (Figures S26 and S27).
  - …for Nuwara Eliya the simulated adult population appears to capture dengue incidence dynamics about two months in advance (Figures S28).

The model outputs when using alternate model's survival curves are comparable in many ways to the outputs when using the primary model's survival curves. In particular, the similarities in the high tercile-high tercile association suggest a robustness in these results. However, there are enough differences in these new model outputs to highlight the sensitivity of this model to the temperature-dependent survival curves. This motivates further work on how to best implement survival curves within such a model.

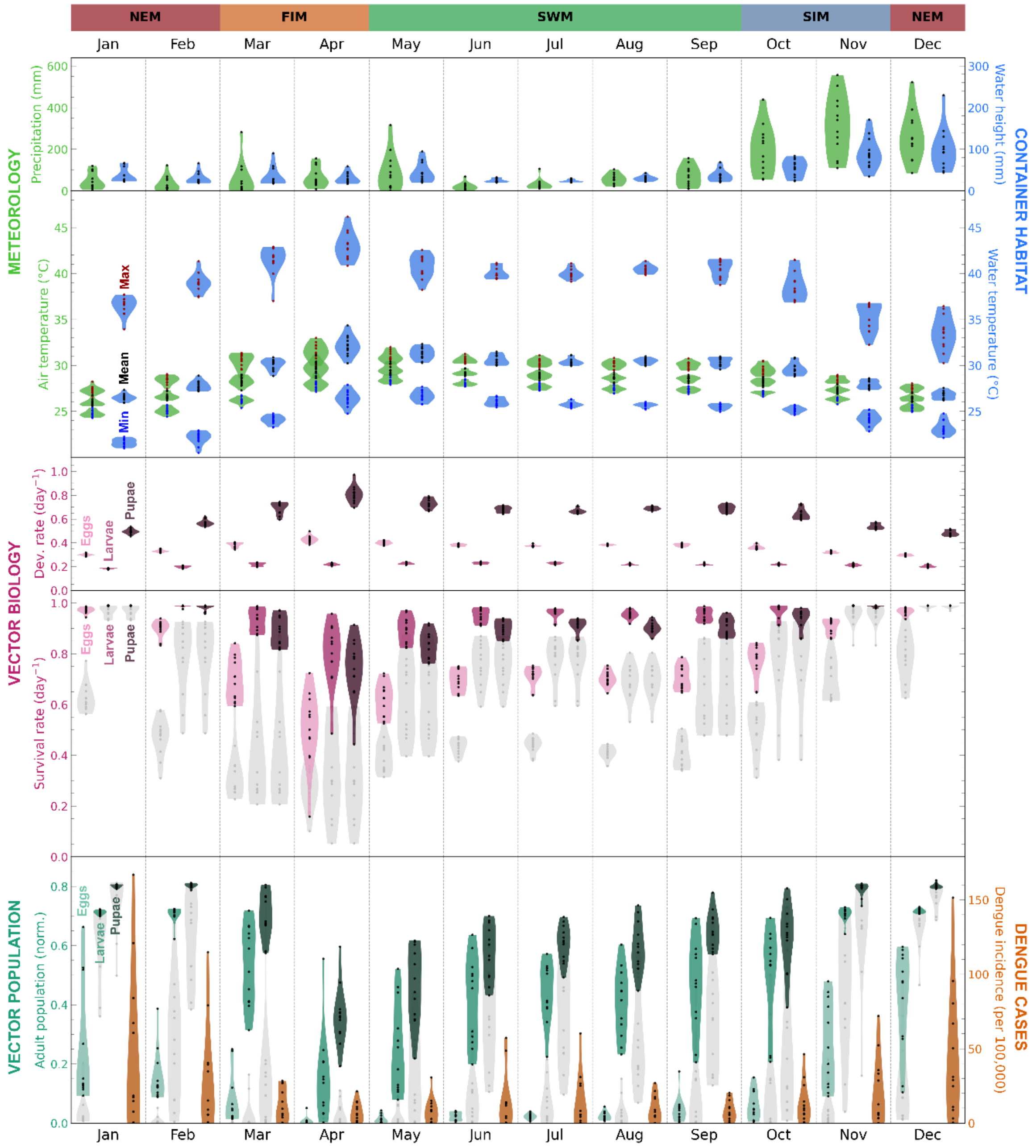

Figure S26. Violin plots of modeling pipeline input/output variables and recorded dengue incidence for **Jaffna** (2007–2020, excluding outbreak years 2017 and 2019). Most of the violin plots correspond to results from using the **alternate temperature-dependent survival model**. In the bottom two panels, gray violin plots correspond to results from using the **primary temperature-dependent survival model**. All variables have been resampled to monthly values such that the violin plot for each calendar month contains one datapoint for each year of data. The variables are as follows: (meteorology) total precipitation, minimum/mean/maximum 2-m air temperature; (container habitat dynamics) mean water height, minimum/mean/maximum water temperature; (vector biology) mean development and survival rate for eggs, larvae, pupae; (vector population dynamics) population of adults from eggs, larvae, pupae at end of month, normalized to initial population. At the top of the figure the months are labeled with Sri Lanka's four monsoonal seasons: NEM (northeast monsoon, Dec–Feb), FIM (first intermonsoon, Mar–Apr), SWM (southwest monsoon, May–Sep), SIM (second intermonsoon, Oct–Nov).

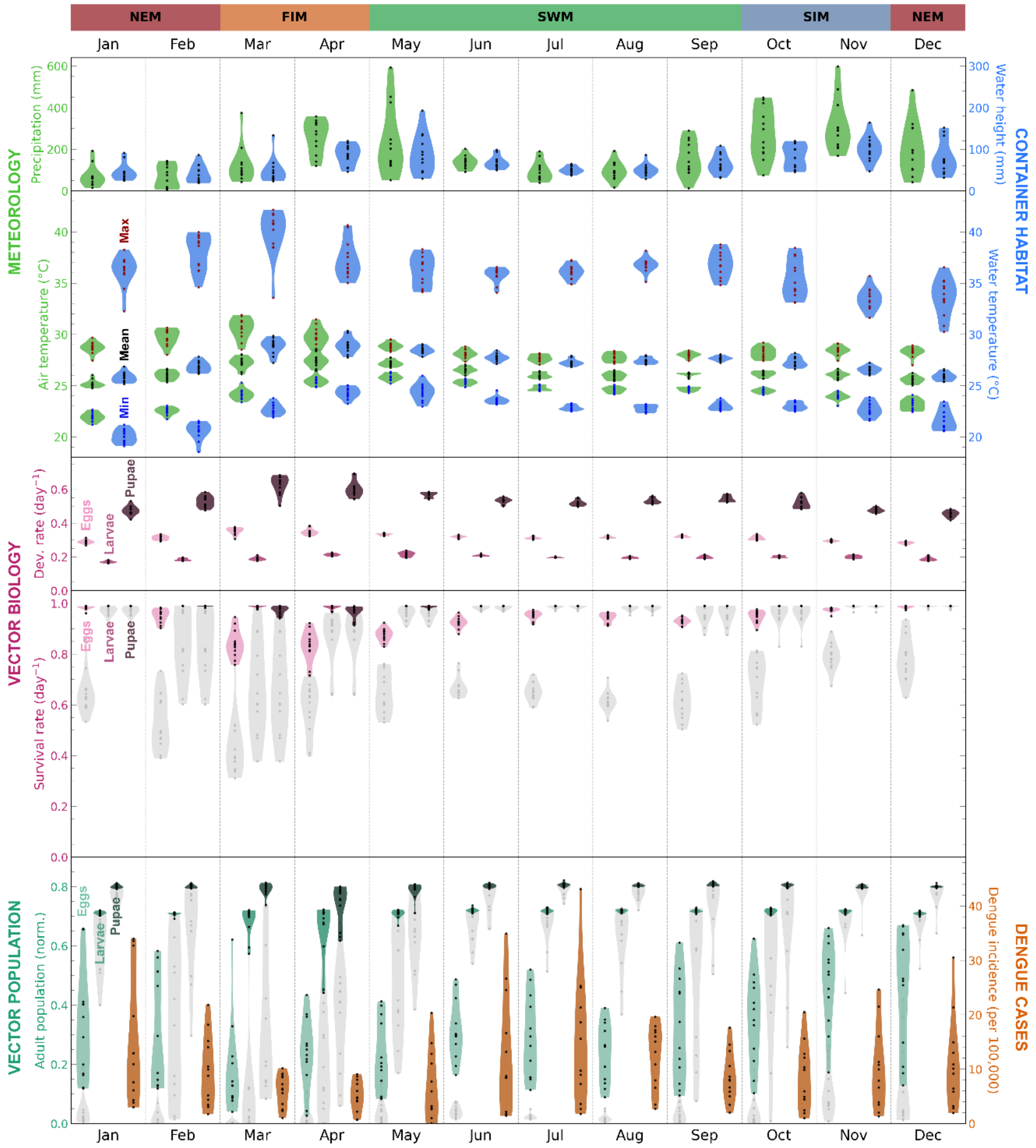

Figure S27. The same as **Figure S26**, but for **Negombo**.

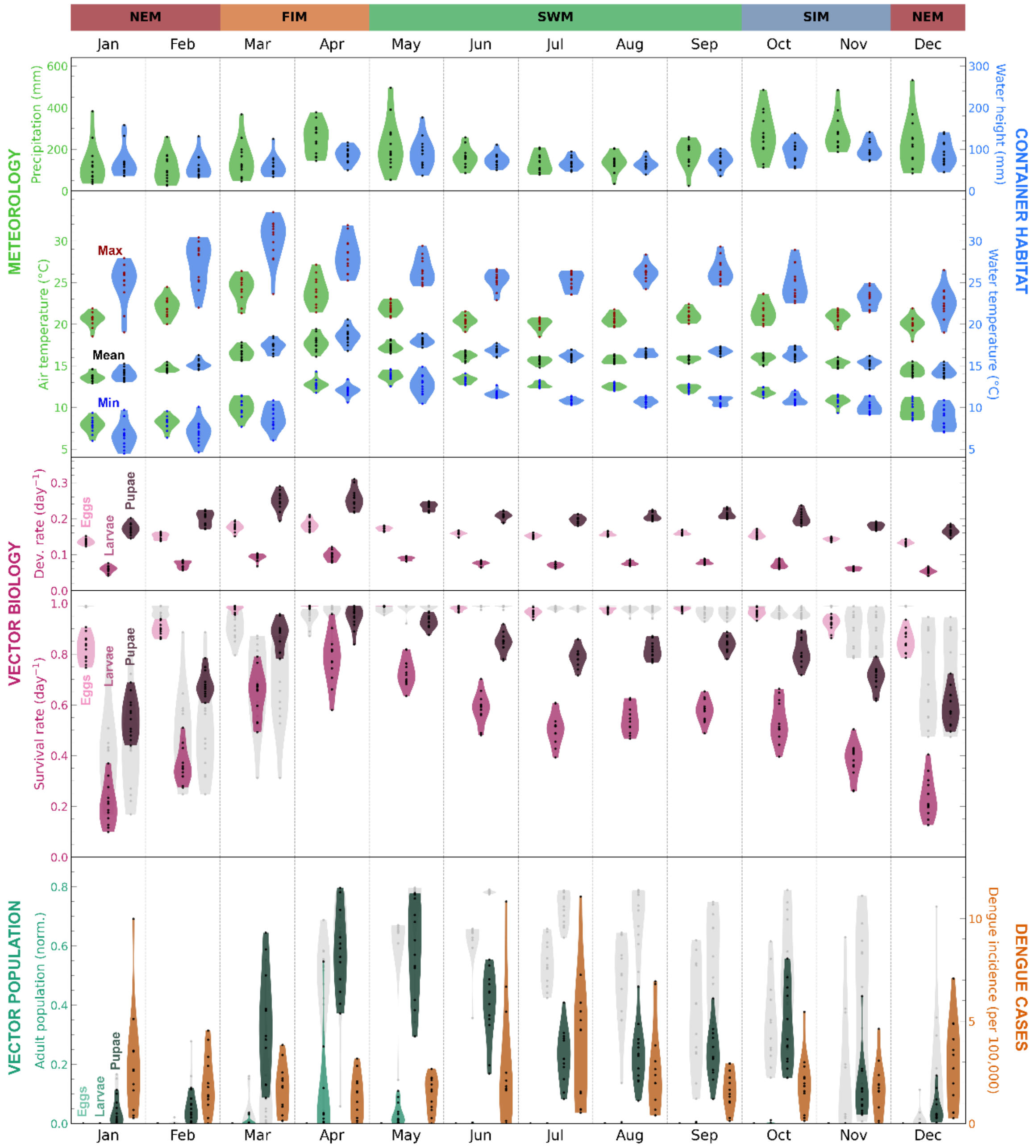

Figure S28. The same as **Figure S26**, but for **Nuwara Eliya**.

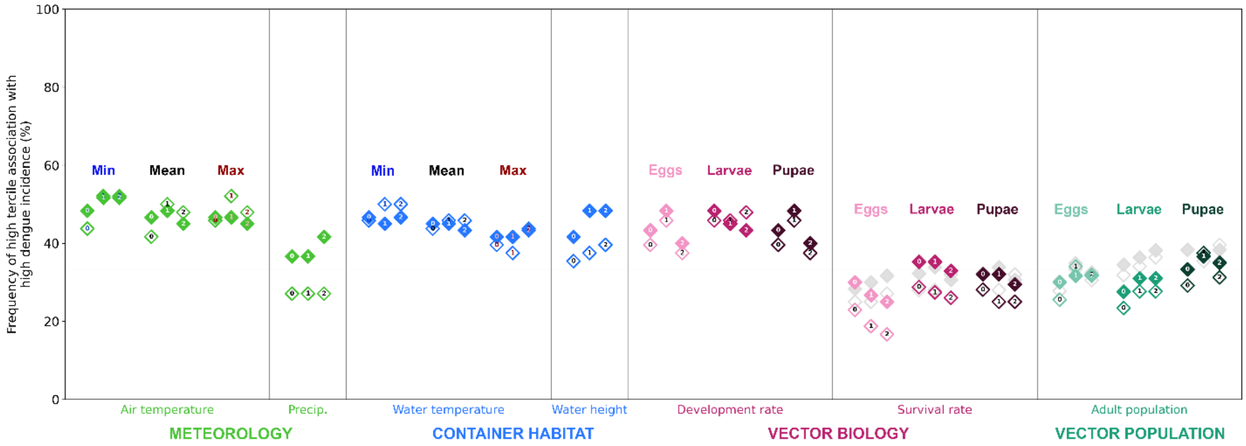

Figure S29. The frequency of association between the high tercile of dengue incidence and the high tercile of each modeling pipeline input/output variable for **Jaffna** (2007–2020). Most of the data points correspond to results from using the **alternate temperature-dependent survival model**. In the bottom two panels, gray data points correspond to results from using the **primary temperature-dependent survival model**. The modeling pipeline variables (all resampled to monthly values) are as follows: (meteorology) minimum/mean/maximum 2-m air temperature, total precipitation; (container habitat dynamics) minimum/mean/maximum water temperature, mean water height; (vector biology) mean development and survival for eggs, larvae, pupae; (vector population dynamics) population of adults from eggs, larvae, pupae at end of month. For each modeling pipeline variable there are six data points corresponding to combinations of different lag times (0, 1, and 2 months; indicated by the number within the data point) and different years of data (including or excluding the outbreak years of 2017 and 2019; indicated by a filled or not filled data point, respectively). The dashed line indicates the expected frequency of association if the associations among terciles were random (33%).

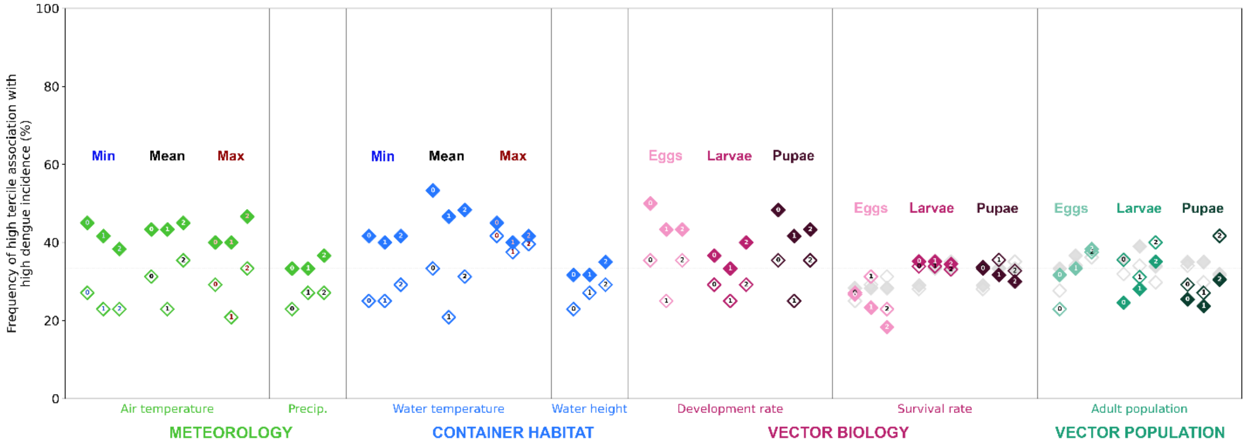

Figure S30. The same as **Figure S29**, but for **Negombo**.

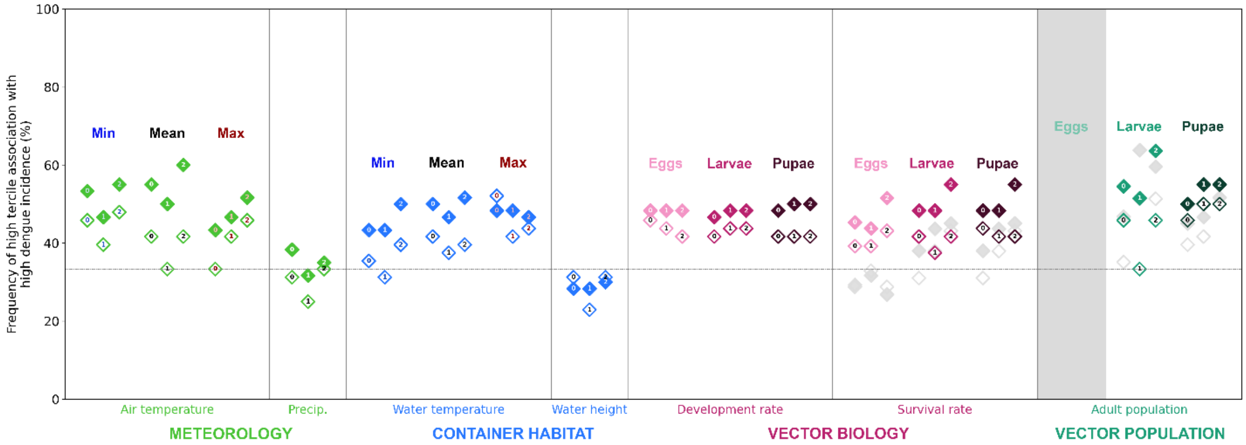

Figure S31. The same as **Figure S29**, but for **Nuwara Eliya**. For Nuwara Eliya the data for population of adults from eggs is omitted, as these values were mostly zero and therefore do not map clearly to terciles.
